# Machine learning framework for predicting ESDD on high-voltage glass insulators with SHAP-based feature optimization

**DOI:** 10.1371/journal.pone.0354892

**Published:** 2026-08-28

**Authors:** Ali Ahmed Salem, Waleed M. Hamanah, Abdullah S. Algamili, Salem Mgammal Al-Ameri, Samir Al-Gailani

**Affiliations:** 1 Interdisciplinary Research Center for Sustainable Energy Systems, Research and Innovation, KFUPM, Dhahran, Saudi Arabia; 2 Department of Electrical Engineering, College of Engineering and Physics, KFUPM, Dhahran, Saudi Arabia; 3 School of Electrical and Electronic Engineering, Universiti Sains Malaysia, Engineering Campus, Nibong Tebal, Penang, Malaysia; 4 Electrical Engineering Department, University of Aden, Yemen; 5 Wireless Communication Centre, Faculty of Electrical Engineering, Universiti Teknologi Malaysia, UTM Johor Bahru, Malaysia; Tishk International University, IRAQ

## Abstract

This paper introduces the use of machine learning (ML) models to predict the severity of pollution on high-voltage outdoor glass insulators using equivalent salt deposit density (ESDD). Four ML models, including Support Vector Machines (SVM), Neural Networks (NN), Extremely Randomized Trees (ERT), and Gaussian Process Regression (GPR), were applied to train 362 data points encompassing fifteen environmental and operational features. Hyperparameter optimization is applied using Bayesian optimization (BO) and random search to enhance model performance. Results show that GPR achieves the highest predictive accuracy, followed by SVM, NN, and ERT across all optimization strategies. SHAP (SHapley Additive exPlanations) was used to identify the most influential features on the performance of the used models based on the training dataset. Models using the top 10 features significantly enhance accuracy and reduce complexity compared to models using the top 5 and 15 features, particularly GPR and ERT models. SHAP-based feature selection and hyperparameter optimization led to the development of efficient, accurate, and interpretable models for predicting contamination severity on high-voltage insulators.

## Introduction

Insulators play a critical role in power transmission lines by providing mechanical support for wires and enhancing electrical insulation [1–4]. This ensures efficient long-distance electricity transmission with minimal energy loss; however, insulator failure can cause transmission line outages [5]. Contamination gradually accumulates on insulator surfaces in the atmosphere, especially in highly polluted areas and coastal regions [6]. Insulators are commonly made from three materials: glass, porcelain, or polymer. Many countries still use glass insulators because they have a longer lifespan, flexible string sizes, and if one disc in the string is damaged, the others remain effective [7]. Despite their advantages, the performance of glass insulators can be diminished by environmental factors such as humidity, fog, temperature, rain, snow, ice, solar radiation, contamination, and others [8–11]. The decrease in performance results from humidity and contamination on the insulator surface, which produce an electrolytic solution that spreads easily across the insulator. This causes the leakage current to increase significantly, potentially leading to flashovers and failure of the insulation system [11,12].

Among the various maintenance strategies for outdoor insulators, periodic washing is the most common method to reduce surface contamination on glass insulators. This process can be done using several techniques; however, modern electric utilities mainly prefer live-line washing methods. These methods use high-pressure water systems mounted on trucks or helicopters, allowing efficient cleaning over large areas without interrupting power supply [13]. A main challenge in insulator maintenance is optimizing the washing schedule. Fixed interval washing doesn’t account for the ever-changing environmental pollution and weather conditions affecting insulators [13]. What works well in one scenario might fail in another, leading to unnecessary maintenance costs or risking system reliability. To improve this, maintenance can benefit from smarter, condition-based strategies. Using advanced models to monitor pollution levels in real time, washing can be scheduled only when necessary, ensuring better system reliability and cost savings [14].

The level of surface contamination prediction on insulators is important for ensuring the efficient operation and maintenance of electrical power systems, where contamination accumulation not only deteriorates the performance of insulators but can also lead to surface discharges and, under severe conditions, flashover issues [7]. The occurrence of such discharges is primarily influenced by the degree of surface contamination, along with environmental factors such as fog, humidity, etc. Because insulators’ leakage current and breakdown voltage exhibit strong sensitivity to these environmental variables, monitoring and analyzing these parameters can provide valuable insights for predicting contamination levels that pose a risk of surface discharges. Consequently, proactive maintenance measures, such as insulator string cleaning, can be strategically implemented to mitigate the risk of flashover, ensuring system reliability and operational safety.

Researchers have conducted several studies to better understand the failure processes of electrical insulators induced by surface contamination, with a focus on analyzing leakage current behavior, impedance-based characterization, and some environmental parameters [7,10,12–28]. Early research focused on determining leakage current under varied pollution levels using both time-domain and frequency-domain studies [22,25,29–32]. Oliveira *et al*. demonstrated that the leakage current harmonics (H7 and H11 harmonics) of insulators under non-uniform pollution emerge as key indicators of contamination [33]. These researchers discovered favorable relationships between leakage current parameters and pollution levels, allowing them to identify essential current measures for maintenance and replacement choices. Simultaneously, researchers have developed statistical models to predict the flashover voltage on insulators, considering pollution intensity and environmental factors like humidity [16,17,20,34,35].

The introduction of machine learning methods has greatly boosted this field. Researchers today have used predictive algorithms to figure out leakage current parameters and flashover voltage based on the environment and have come up with classification models for finding out how healthy an insulator is in a range of operating conditions [14,18,26,36–40]. Furthermore, some studies have explored the use of leakage current and flashover voltage data in combination with statistical techniques to evaluate pollution levels, paving the way for predictive maintenance strategies [27,41,42]. Although we have well-documented, established pollution limits, additional research is required to identify the contamination levels that trigger surface discharges and subsequent flashovers. This data could enhance the scheduling and optimization of insulator cleaning, guaranteeing the timely execution of maintenance. Standard statistical methods and intelligent machine learning algorithms have used various leakage current characteristics, environment parameters, and flashovers to examine insulator failures. Popular algorithms include artificial neural networks [43], fuzzy logic systems [44], support vector machines [12,45], decision trees [46], logistic regression [47], and simpler statistical approaches [48]. The recent works on pollution level (ESDD) prediction using machine learning algorithms are summarized and elaborated in Table 1. Despite these developments, there is still a need to create an optimized, resilient, and highly accurate computational framework with finely tuned hyperparameters. Such a model would offer accurate projections of pollution levels, allowing for proactive measures to minimize surface discharges and reduce the danger of flashovers.

**Table 1:** Represented machine learning algorithms for pollution severity (ESDD) prediction.

Reference	Year	Models/Opt.	Features	Remark
[12]	2024	MLP and SVR	Leakage current characteristics: max value, RMS, 1st harmonic, 3rd harmonic, 5th harmonic and THD	An accuracy of 99.8% for the MLP and 97.1% for the SVM model
[48]	2024	ANN	Electric field characteristics: K3, αTHD, RMS value, Peak value	The models achieved an accurate range of 90% − 95%
[49]	2023	RF and MLR	Hyperspectral lines and colour components	The models achieved an accurate range of 84%−93.75%
[26]	2017	RF, SVM	Deposition time, Hydrophobicity, Particle size, Altitude, Distance from coastline, Rainfall, Temperature, Wind velocity, Material, Position factor, Surface area, Surface orientation, Total length, Voltage type, Voltage level, Polarity	The models achieved an accuracy of 95.1% for RF and 86% for the SVM model
[50]	2013	ANN	Leakage current characteristics: mean value, max value, standard deviation.	The models achieved an accurate range of 87% − 91%
[36]	2011	BNN and ANFIS	Leakage current characteristics: mean value, max value, standard deviation, THD	An accuracy of 94.3% for BNN, 99.8% for the ANFIS
[51]	2010	BP ANN	Leakage current characteristics: mean value, max value, standard deviation, applied voltage, and humidity	An accuracy range of 90% − 93%
[52]	2025	IHHO-XGBoost	Temperature, humidity, wind speed, solar radiation, elevation, dew point, and rainfall	The models achieved an accuracy of 99.3%
This work	2026	[SVM, NN, ERT, and GPR]/[RS, BO]	Listed in Table 2	The models achieved an accurate range of 98.0% − 99.9%

This study aims to develop a reliable data-driven framework for predicting the contamination severity of glass insulators using real-world environmental and electrical data. The novelty of this work lies in the integration of geographically diverse field measurements with optimized machine learning models. Multiple location-based datasets are constructed to capture spatial variability, and different feature subsets (15, 10, and 5 features) are systematically evaluated to analyze the impact of feature selection on prediction performance. Furthermore, both Random Search and Bayesian Optimization are employed to enhance model accuracy. Feature importance analysis is also conducted to identify the most influential parameters affecting contamination severity, providing both predictive capability and physical insight into the problem. The main contributions of this paper are briefly discussed below:

1) Glass insulators were collected from different geographical regions considering their distance from the sea and operational lifetime. In addition, key meteorological parameters, including humidity, pressure, rainfall, temperature, solar radiation, wind speed, and wind direction, were systematically recorded at the ESDD measurement sites.
2) The pollution severity and electrical characteristics of the collected insulators were experimentally evaluated. Statistical analyses were then performed to investigate the relationships between these characteristics and contamination severity. Based on the insulator locations, three distinct datasets were constructed to capture spatial variability and provide effective inputs for the proposed models.
3) A data-driven framework based on multiple machine learning models (MLP, ERT, GPR, and SVM) was developed to predict the contamination severity of 33 kV glass insulator strings. Hyperparameter tuning was conducted using Random Search and Bayesian Optimization, where the average RMSE obtained from 5-fold cross-validation was used as the optimization objective to ensure robust model performance.
4) Finally, the importance of input features was evaluated to identify the most influential environmental and operational parameters affecting contamination severity and to enhance the interpretability of the developed models.

## Materials and methods

### Study Parameters

**Fig 1:**
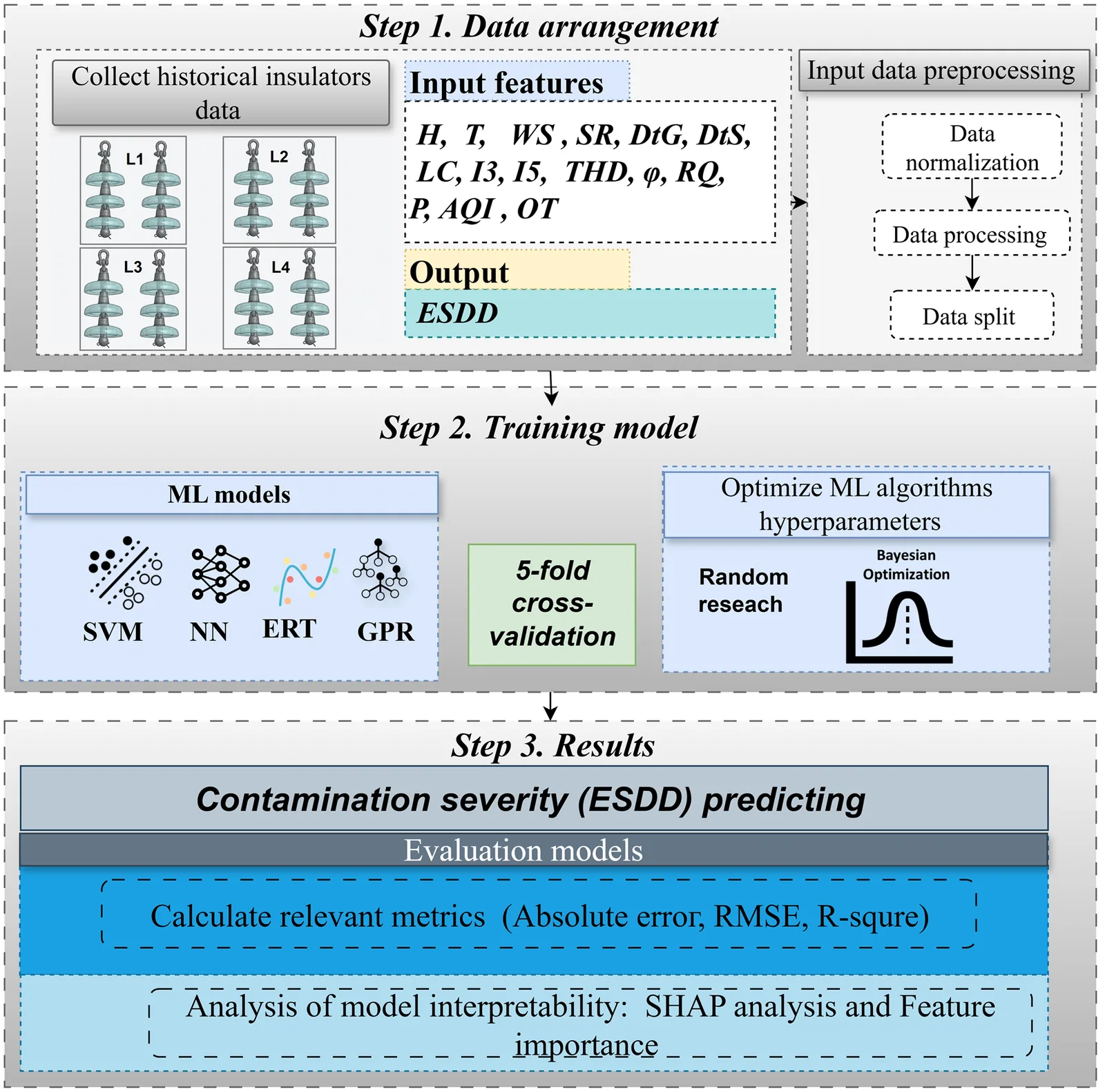
Contamination severity prediction methodology.

In this work, we have compiled an extensively curated dataset for 8 samples of 33 kV insulator strings for four different locations (2 strings in each location). The chosen four locations are suffering from the rapid buildup of salt contamination, as they are near the seacoast at approximately 1–9 km. We measured the meteorological parameters, including relative humidity (H), temperature (T), wind speed (WS), Solar radiation (SR), distance to ground (DtG), distance to sea (DtS), quantity of rainfall (QR), operating period (OP), and air quality index (AQI), corresponding to the period of ESDD measurements (33 weeks). These measurements were taken at regular intervals to ensure accuracy and to capture any variations throughout the day. Analyzing these parameters will help us understand their potential impact on observed environmental conditions during the study period. Meanwhile, the electrical properties, including leakage current (LC) and phase shift angle between current and applied voltage (φ) of the insulator strings, were measured. Given the strong correlation between pollution severity and the LC odd harmonics [31], we extracted the third and fifth harmonics for use as features. The leakage current and flashover voltage of the selected samples were tested in the laboratory after confirming that the pollution stayed on their surface and was not affected by transfer. The glass insulator strings were tested separately from the power network for safety and to avoid any interruptions to the network. The strings were energized by 33 kV AC, and the leakage current was measured using a CT sensor. The phase shift angle φ, third harmonic I3, and fifth harmonic I5 were extracted from the original leakage current signal using MATLAB software. The non-soluble deposit density measurement is performed. The framework of contamination prediction in this study is presented in Fig 1. The literature shows that the deposited ESDD rate can effectively evaluate the condition of insulators. After examining numerous potential factors that influence ESDD, this research concentrates on empirically studying the most significant ones.

**Fig 2:**
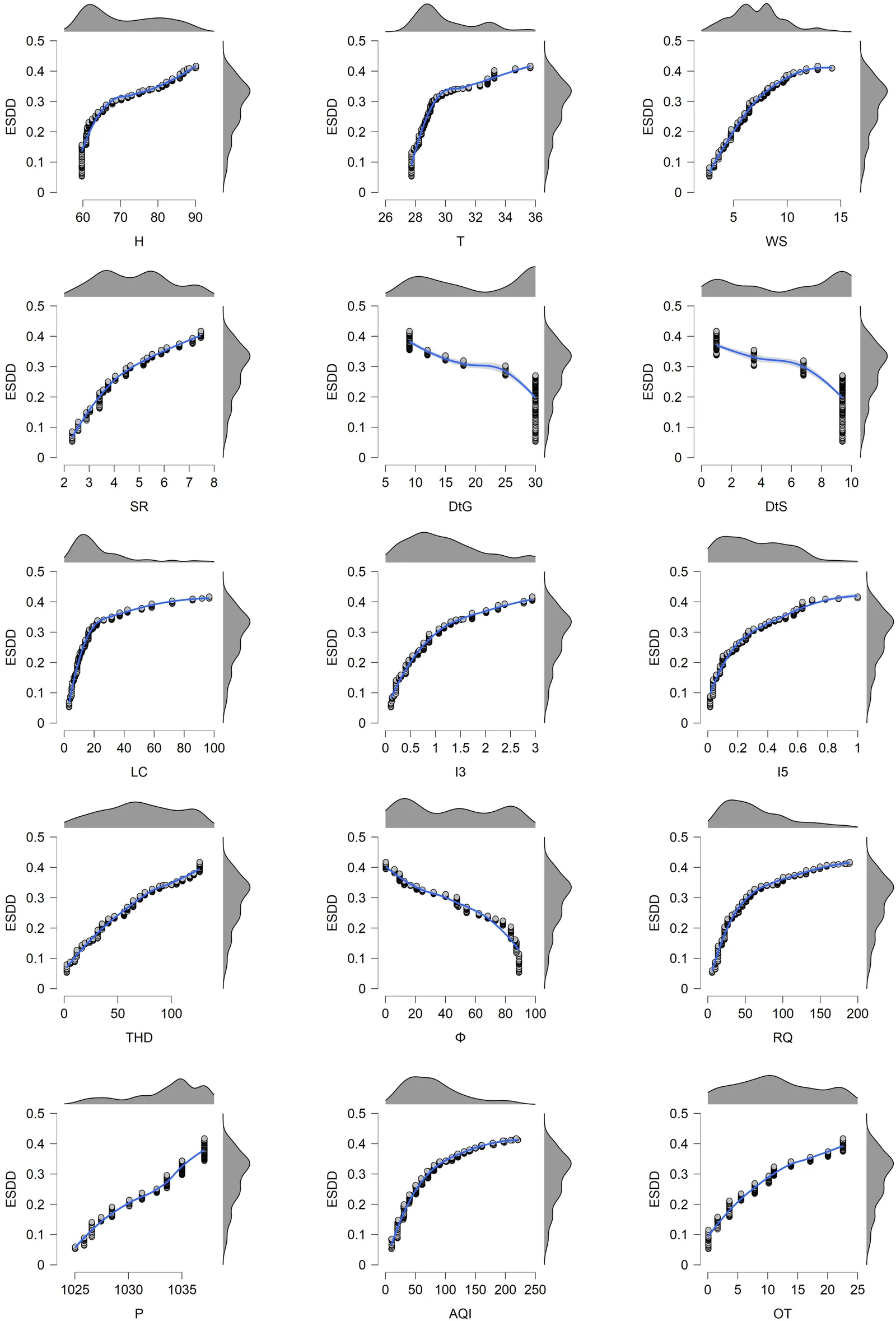
Correlation ESDD with inputs.

Fig 2 demonstrates how the ESDD on the insulator surface relates to measured factors over 36 weeks. According to Fig 2, of the fifteen characteristics considered, twelve (H, T, WS, SR, LC, I3, I5, THD, RQ, OT, AQI) exhibit a direct proportional relationship with ESDD; ESDD increases as these variables increase. Conversely, ESDD has an inverse relationship with DtS, DtG, and Φ.

### ESDD-based insulator pollution severity

Standards such as IEC 60815−1 [53], IEC 60507 [54], and IEEE [55] were used to quantify the ESDD. ESDD measurement seeks to determine, in mg/cm2, the concentration of soluble conductive impurities on an insulator’s surface. To carry out the ESDD measurement technique, the insulator must be carefully removed from the implanted site without damaging the contaminated layer [53]. To remove the contaminated layer, the person in charge should use a cotton pad, sponge, or brush along with up to 500 cm3 of distilled water and protective gloves. The sample’s ESDD can be calculated as:


$$\begin{array}{c} ESDD=S(a)\times \left(V_{w}.\overset{-\text{1}}{\underset{in}{A}}\right) \end{array}$$ (1)

where *V*_*w*_ represents the volume of water utilized in cm^3^, *A*_*in*_ denotes the insulator surface area on which the pollutants were collected in cm^2^, and *S(a)* indicates the salinity in kg/m^3^. According to [56], during two months, the insulator contamination level in certain coastal locations reaches an ESDD threshold higher than 0.1 mg.cm^-2^, necessitating special focus to estimate and mitigate their impact on the power system.

### Data Preprocessing

In this study, 362 data points of ESDD were collected from four different locations. Table 2 shows the data description, and Table 3 provides statistics and limitations of the selected features. Notably, a more detailed description of measurement replication and uncertainty has been included to ensure data reliability. At each experimental location, multiple insulator samples were monitored, with two samples per site. For each sampling event, ESDD measurements were conducted once because the insulator surface needed to be cleaned of pollution according to standard procedures. Before ESDD measurement, the leakage current measurements were recorded using calibrated sensors with a specified accuracy of ± 0.1 mA, and repeated measurements showed consistent behavior with minimal deviation. Leakage current was measured in 5 replicates under the same pollution (ESDD) level following standard procedures to ensure repeatability, and the average value was used for analysis. To evaluate repeatability, selected measurements were repeated under identical conditions, and the observed variation remained within acceptable limits, indicating good measurement stability. The overall uncertainty associated with the measurement process is therefore considered low, and the dataset is deemed representative of actual environmental and operational variability rather than measurement noise. All data preprocessing steps, including normalization, feature ranking, and feature subset selection, were performed strictly within the training data for each scenario and for every repetition to prevent data leakage. The learned parameters and selected feature subsets were then applied to the corresponding validation and test sets. This fold-wise procedure ensures consistency with the SHAP analysis and maintains the integrity and generalizability of the model evaluation.

**Table 2:** Summary of experimental locations and data collection details.

Experiment location	4 locations (L1,L2,L3, and L4)
Insulators sample number	2 string insulator samples for each location
Data collection period	33 weeks
Data collected from L1 (Dts = 1 km)	101 data points (50 for string 1 and 51 for string 2); some weeks we measure once and some weeks twice, over 33 weeks
Data collected from L2 (Dts = 3.5 km)	58 data points (33 for string 1 and 25 for string 2)
Data collected from L3 (Dts = 6.8 km)	54 data points (33 for string 1 and 21 for string 2)
Data collected from L4 (Dts = 9.4 km)	149 data points (99 for string 1 and 50 for string 2); string 1 was measured 3 times weekly.
Total data collected	362 data points

**Table 3:** Variable’s characteristics.

Feature	Unit	Mean	Std.	Skewnes	Kurtosis	Min	Max	25th %	50th %	75th%
***H***	%	71.14	9.641	0.4	−1.259	59.72	90.137	61.78	68.86	80.17
***T***	˚C	30.07	2.025	1.033	0.109	27.74	35.654	28.64	29.21	31.15
***WS***	m/s	7.161	2.371	0.495	0.237	2.74	14.185	5.665	6.78	8.215
***SR***		4.831	1.423	0.209	−0.888	2.321	7.468	3.756	4.649	5.883
***DtG***	m	20.77	8.787	−0.135	−1.729	9	30	12	21.5	30
***DtS***	km	5.723	3.576	−0.232	−1.665	1	9.4	1	6.8	9.4
***LC***	mA	24.37	20.59	1.758	2.681	3.328	96.71	10.72	16.75	31.67
***I3***	mA	1.17	0.736	0.771	−0.068	0.111	2.936	0.648	1.068	1.558
***I5***	mA	0.325	0.221	0.58	−0.225	0.016	0.999	0.146	0.271	0.476
***THD***	%	71.59	35.26	−0.078	−0.961	2.41	126.44	46.92	70.64	100.3
***Φ***	˚	44.09	31.90	0.298	−1.239	0.211	100.1	12.53	40.1	68.20
***RQ***	m3/h	63.45	42.71	1.01	0.419	5.738	189.11	32.23	54.11	92.93
***P***	KPas	1033	3.361	−0.839	−0.312	1025.	1037.1	1031	1035	1035.1
***AQI***		79.52	47.24	0.954	0.564	9.656	221.15	40.50	71.35	101.6
***OT***	year	11.33	6.771	0.165	−0.977	0.098	22.561	5.561	11.1	17.09
***ESDD (out)***	mg/cm^2^	0.126	0.076	0.65	−0.282	0.011	0.338	0.065	0.114	0.173

Models implemented in Python utilized libraries like Keras, Scikit-learn, and TensorFlow, trained on a system with a 12th Gen Intel® Core™ i7-12700 processor and an NVIDIA GeForce RTX 3060 GPU. The study focuses on assessing the correlation between meteorological factors and electrical parameters affecting insulator contamination severity, using Pearson’s correlation coefficient (r) equation (2) to identify significant indicators for model input and reduce unimportant features. The correlation values for most features are high, between 0.73 and 0.82, with some feature correlations reaching 0.99. Although strong multicollinearity exists among several predictors, it does not adversely affect the predictive performance of tree-based ensemble models; therefore, no feature elimination was performed. The models considered in this study belong to different methodological classes. ERT is a tree-based ensemble method, SVM is a margin-based kernel method, NN is a neural-network-based model, and GPR is a probabilistic kernel-based model. Therefore, the effect of correlated input variables should not be interpreted using tree-based reasoning for all models. While tree ensembles such as ERT may naturally handle correlated predictors through recursive split selection, the remaining models address correlation through their respective kernel, representation-learning, or probabilistic formulations. Nevertheless, predictive performance may remain strong even when inputs are correlated, although the interpretation of this robustness differs across model types. Unlike linear models, these algorithms do not rely on coefficient estimation and are therefore not adversely affected by correlated predictors in terms of predictive performance. During training, the models naturally select splits among correlated variables, effectively handling redundancy without degrading accuracy. However, such redundancy may influence the distribution of feature importance and should be considered when interpreting model explanations.


$$\begin{array}{c} =\frac{\sum_{i=\text{1}}^{n}\left(X_{i}-\overset{―}{X})(Y_{i}-\overset{―}{Y}\right)}{\sqrt{\sum_{i=\text{1}}^{n}(X_{i}-\overset{―}{X}{)}^{\text{2}}(Y_{i}-\overset{―}{Y}{)}^{\text{2}}}} \end{array}$$ (2)

where $\overset{―}{X}$and$\overset{―}{Y}$ denote the means of the variables X and Y, and n represents the observations (data points). The r ranges from −1–1, where a value of 1 indicates the highest positive linear association, 0 signifies the absence of a linear relationship, and −1 represents the highest negative linear association between variables x and y.

**Fig 3:**
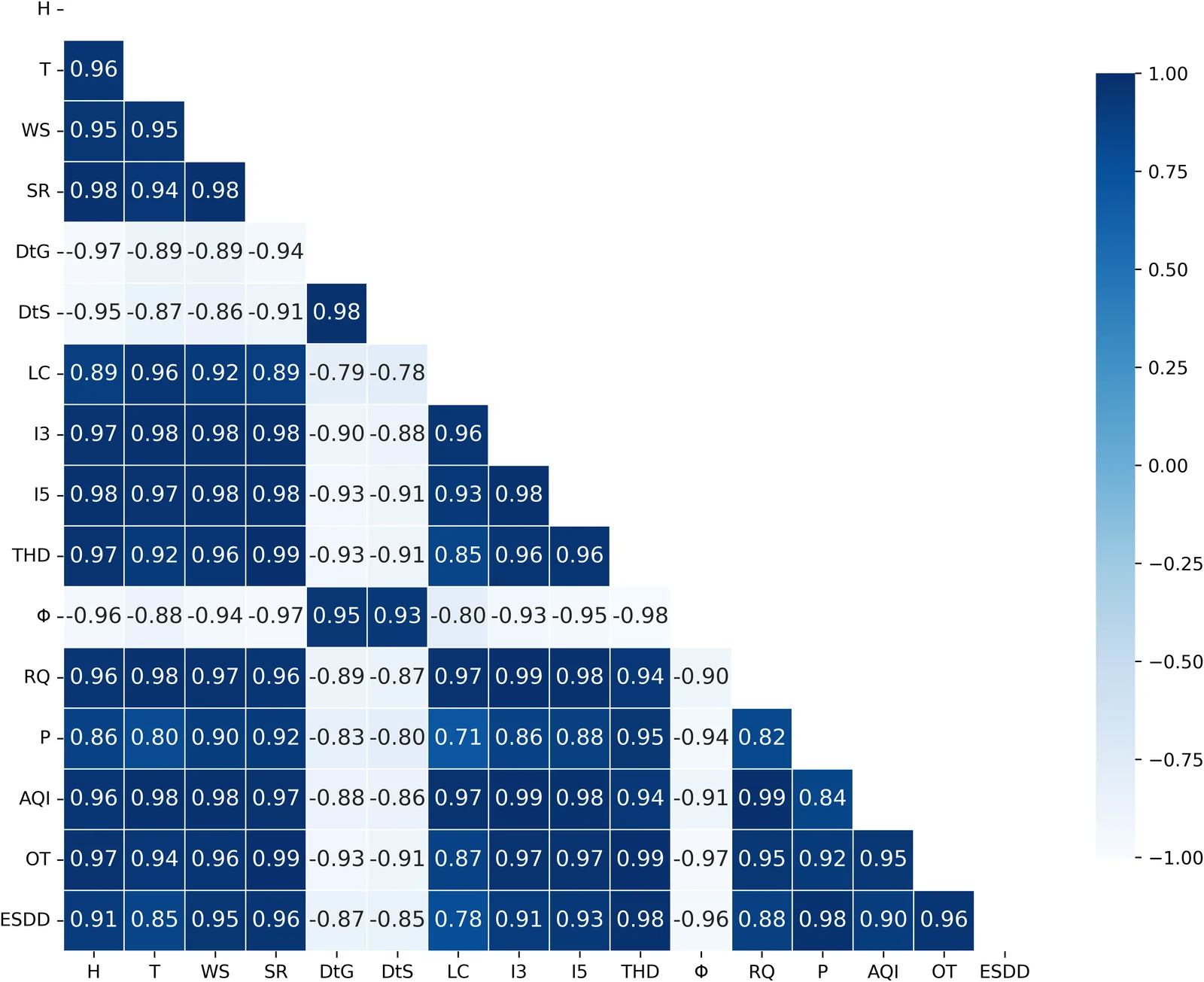
Correlation matrix of the input features and the output variable.

**Fig 4:**
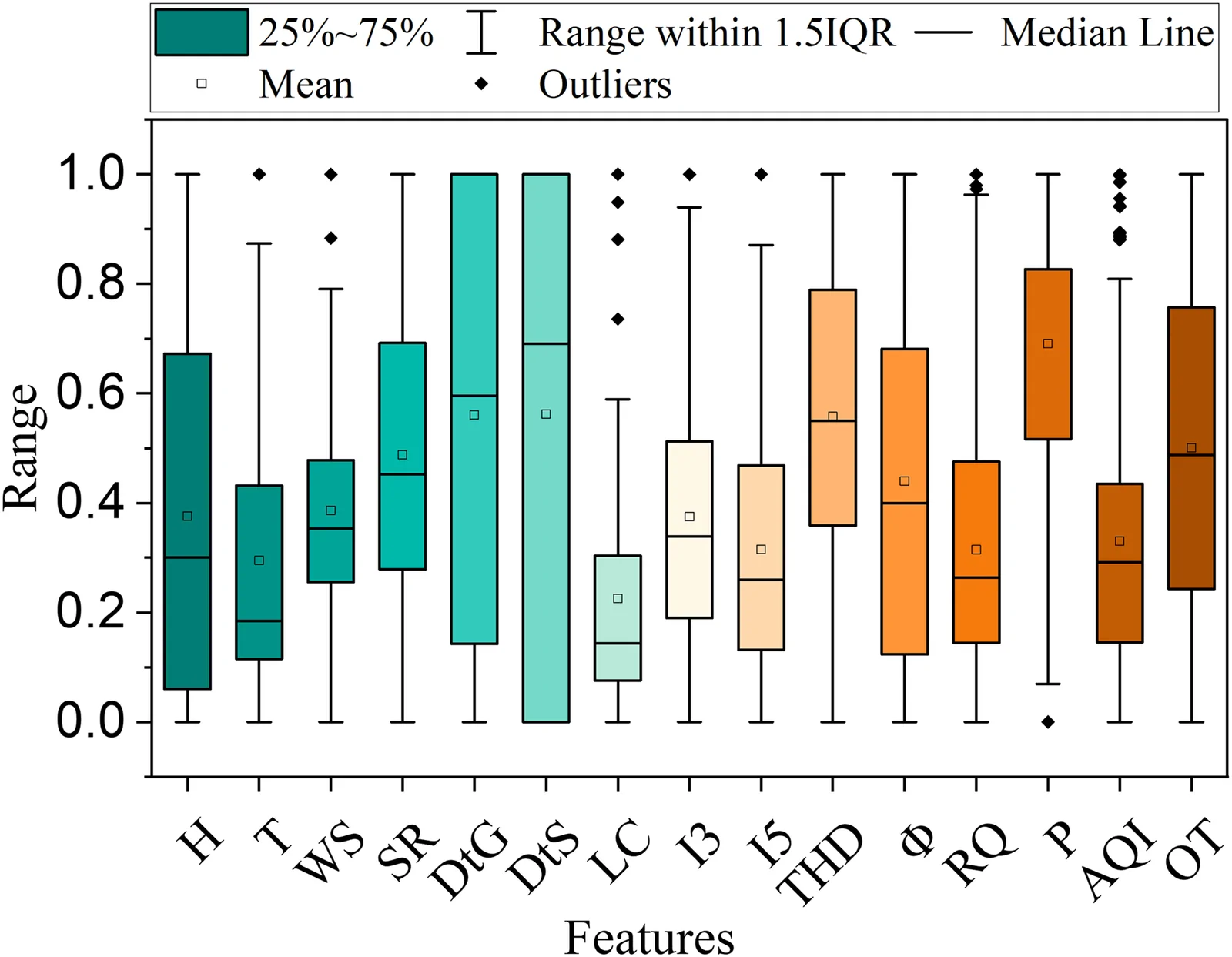
Min-max normalization box plot of pollution parameters.

Fig 3 shows the correlation matrix of the selected features and ESDD. To prevent gradient explosions and enhance the learning efficiency of the proposed ML models, handling raw data with a broad value range is essential. Inadequate pre-processing can hinder model fitting and regression, thereby compromising overall prediction accuracy. Consequently, data pre-processing is a critical step that includes standardization, normalization, imputing missing values, and removing irrelevant entries (null values). Specifically, min-max normalization is applied to scale data within the [0,1] range, effectively reducing training time and minimizing regression error while preserving the intrinsic relationships among data points. The min-max normalization technique is mathematically represented in (5).


$$\begin{array}{c} x_{v}=\frac{x_{i}-x_{min}}{x_{max}-x_{min}} \end{array}$$ (3)

where *x*_*min*_ and *x*_*max*_ denote the minimum and maximum values of variable *x*, respectively, and *x*_*i*_ is the ith value of *x*. Fig 4 shows a box plot of pollution prediction characteristics after min-max normalisation.

### Proposed Machine Learning models

**Fig 5:**
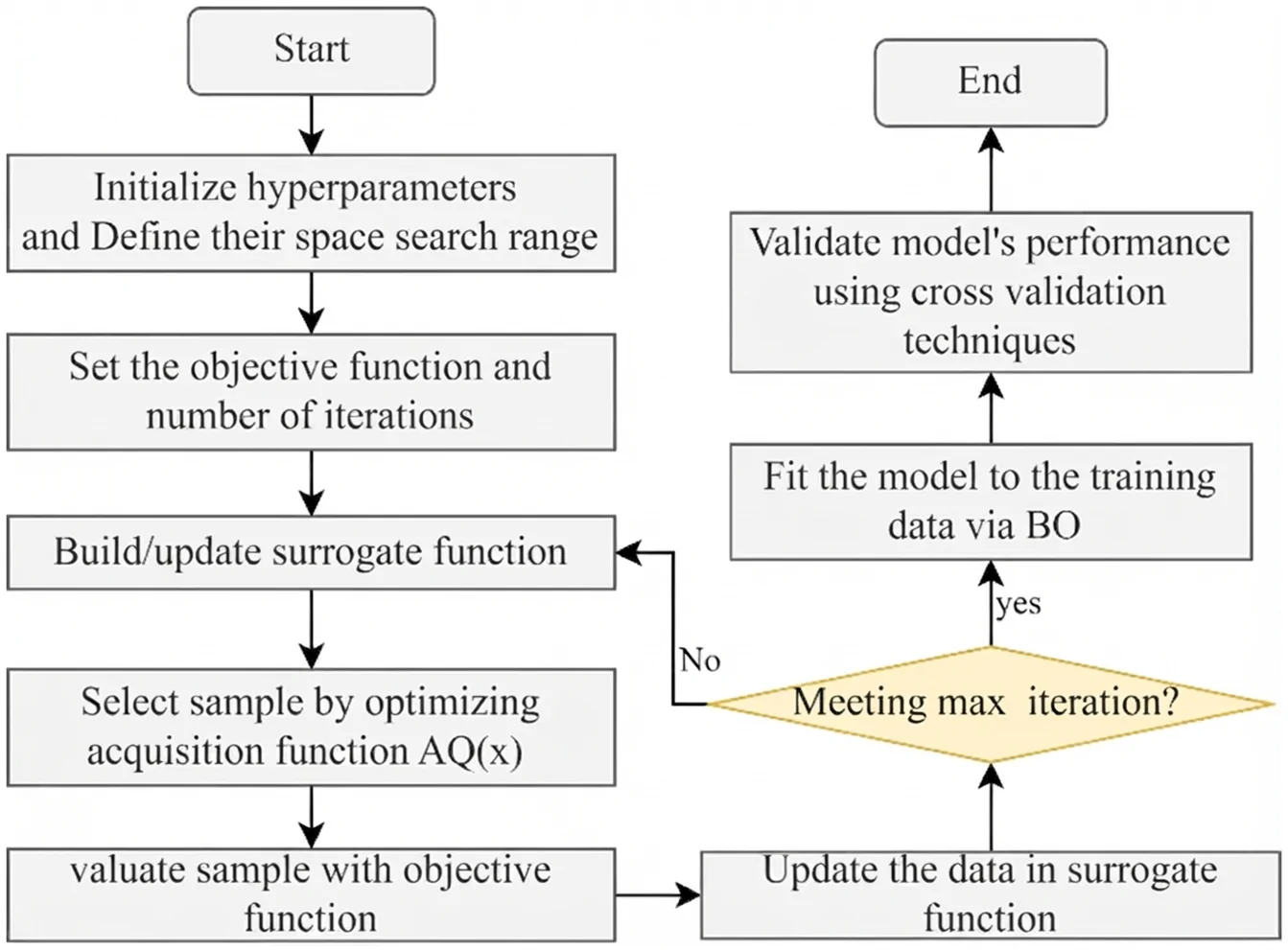
Flowchart of Bayesian optimization.

The study integrates multiple machine learning models and optimization strategies to predict Equivalent Salt Deposit Density (ESDD) by capturing complex nonlinear relationships among environmental variables. Support Vector Machines (SVM), Neural Networks (NN), Ensemble of Regression Trees (ERT), and Gaussian Process Regression (GPR) were selected to represent diverse learning paradigms with complementary strengths. Neural Networks (NN) are employed to model nonlinear dependencies through layered neuron structures and activation functions, with parameters optimized via backpropagation. In addition, ensemble learning techniques, including bagging, boosting, and Extremely Randomized Trees (ERT), are used to improve predictive accuracy and robustness. These tree-based methods effectively handle complex feature interactions and are less sensitive to multicollinearity, making them suitable for this application. Gaussian Process Regression (GPR) [57,58] is also utilized as a probabilistic approach, providing both predictions and associated uncertainty by modeling data through kernel-based covariance functions. This enhances the reliability of the predictions, particularly in cases with limited or noisy data. To optimize model performance, hyperparameter tuning is conducted using Random Search and Bayesian Optimization. While Random Search explores the space more efficiently, Bayesian Optimization (Fig 5) leverages a probabilistic surrogate model to identify optimal configurations with fewer evaluations. For brevity, the detailed mathematical formulations, algorithmic steps, and parameter settings of these models are provided in the **Supporting information File**.

The models were compared based on predictive accuracy, generalization performance, and computational efficiency. Results indicate that ensemble-based (ERT) and probabilistic (GPR) approaches provided more stable predictions, while NN achieved strong performance in capturing complex patterns. SVM offered competitive results with relatively lower computational cost. Overall, the comparison highlights the trade-offs between accuracy, robustness, and computational demand across different modeling approaches.

## Results and discussion

This section evaluates the effectiveness of four proposed machine learning algorithms in determining ESDD using reduced error, training time, and model sizes. It discusses the influence of hyperparameters and how BO and RS optimizers improve performance. For training and testing, an 8:2 split was used, and a 5-fold cross-validation method was utilized. For hyperparameter optimization, 30 iterations were chosen. Table 4 shows the optimized hyperparameter values for all ML proposed techniques using both optimizers, BO and RS.

**Table 4:** The proposed models optimized hyperparameters.

Algorithm	Hyperparameters	Range	RS	BO
SVM	Epsilon	8.6446e-058.6446	3.25E-04	6.2E-05
	Box constraint	0.001-1000	26.56	157.83
	Kernel function	Gaussian, Linear, Quadratic, Cubic	Linear	Gaussian
	Kernel scale	0.001-1000	–	1.7655
	Standardize data	true, false	true	true
NN	No. Fully connected layers	1	1	1
	First layer size	10	10	10
	Activation	ReLU	ReLU	ReLU
	Regularization strength (λ)	0-10	0.089	1.932
ERT	Ensemble method	Bagging, LSBoost	Bagging	LSBoost
	Min. leaf size	1-181	43	127
	Number of Learners	10-500	150	168
	Learning rate	0.001-1	0.001	0.1975
GPR	Sigma	0.0001-0.8137	1.08E-04	1.693E-04
	Basis function	Constant, Zero, Linear	Linear	Linear
	Kernel scale	0.21149-211.4915	30.638	15.48
	Kernel function	NE, NM3/2, NM 5/2,	NM 5/2	NM 3/2
	Standardize	true, false	true	true

For evaluating the accuracy and performance of the proposed model In this study, the data were split into four scenarios:

- **Scenario 1**: Blocked Time Split ((1–26) weeks for training and (27–33) weeks to testing), as shown in Table 5.
- **Scenario 2**: Leave-One-Location (Training for selected location (L1, L2, L3) and testing in other location (L4)), as shown in Table 6.
- **Scenario 3**: Combined: Leave-One-Location + Time-Blocked: Train on early weeks from other locations and test on later weeks from an unseen location, as shown in Table 7.
- **Scenario 4**: Random data distribution and split data 80/20.

**Table 5:** Hierarchy table of data splitting according to Blocked Time Split (Scenario 1).

Location	String ID	Weeks No.	Sampling event	Measure No.	Replicate rate*	Interval time (days)	ESDD Cleaning/week	Split assignment
L1	S1A.	W1-W26	E001–E040	40	1.54	4	Yes	Training
	S1B.	W1-W26	E001–E042	42	1.62	4	Yes	Training
L2	S2A.	W1-W26	E001–E026	26	1.00	7	No	Training
	S2B.	W1-W26	E001–E020	20	0.77	9	No	Training
L3	S3A.	W1-W26	E001–E026	26	1.00	7	No	Training
	S3B.	W1-W26	E001–E015	15	0.58	12	Yes	Training
L4	S4A.	W1-W26	E001–E080	80	3	2	Yes	Training
	S4B.	W1-W26	E001–E040	40	1.54	4	Yes	Training
L1	S1A.	W27-W33	E0040–E050	10	1.43	5	Yes	Testing
	S1B.	W27-W33	E0042–E052	10	1.43	5	Yes	Testing
L2	S2A.	W27-W33	E0026–E033	7	1.00	7	Yes	Testing
	S2B.	W27-W33	E0020–E026	6	0.86	8	No	Testing
L3	S3A.	W27-W33	E0026–E033	7	1.00	7	Yes	Testing
	S3B.	W27-W33	E015–E021	6	0.86	8	Yes	Testing
L4	S4A.	W27-W33	E080–E099	19	2.71	3	Yes	Testing
	S4B.	W27-W33	E040–E050	10	1.43	4	Yes	Testing

Replicate rate* = Measure No./Weeks No. Note. A replicate rate >1.5 means the region has high pollution depositions

**Table 6:** Hierarchy table of data splitting according to Location (Scenario 2).

Location	String ID	Weeks No.	Sampling event	Measure No.	Replicate rate*	Interval time (days)	ESDD Cleaning/week	Split assignment
L1	S1A.	W1-W26	E001–E040	40	1.54	4	Yes	Training
	S1B.	W1-W26	E001–E042	42	1.62	4	Yes	Training
L2	S2A.	W1-W26	E001–E026	26	1.00	7	No	Training
	S2B.	W1-W26	E001–E020	20	0.77	9	No	Training
L3	S3A.	W1-W26	E001–E026	26	1.00	7	No	Training
	S3B.	W1-W26	E001–E015	15	0.58	12	Yes	Training
L4	S4A.	W1-W26	E001–E080	80	3	2	Yes	Testing
	S4B.	W1-W26	E001–E040	40	1.54	4	Yes	Testing
L1	S1A.	W27-W33	E0040–E050	10	1.43	5	Yes	Training
	S1B.	W27-W33	E0042–E052	10	1.43	5	Yes	Training
L2	S2A.	W27-W33	E0026–E033	7	1.00	7	Yes	Training
	S2B.	W27-W33	E0020–E026	6	0.86	8	Yes	Training
L3	S3A.	W27-W33	E0026–E033	7	1.00	7	Yes	Training
	S3B.	W27-W33	E015–E021	6	0.86	8	Yes	Training
L4	S4A.	W27-W33	E080–E099	19	2.71	3	Yes	Testing
	S4B.	W27-W33	E040–E050	10	1.43	4	Yes	Testing

**Table 7:** Hierarchy table of data splitting according to Combined: Leave-One-Location + Time-Blocked (Scenario 3).

Location	String ID	Weeks No.	Sampling event	Measure No.	Replicate rate*	Interval time (days)	ESDD Cleaning/week	Split assignment
L1	S1A.	W1-W26	E001–E040	40	1.54	4	Yes	Training
	S1B.	W1-W26	E001–E042	42	1.62	4	Yes	Training
L2	S2A.	W1-W26	E001–E026	26	1.00	7	No	Training
	S2B.	W1-W26	E001–E020	20	0.77	9	No	Training
L3	S3A.	W1-W26	E001–E026	26	1.00	7	No	–
	S3B.	W1-W26	E001–E015	15	0.58	12	Yes	–
L4	S4A.	W1-W26	E001–E080	80	3	2	Yes	–
	S4B.	W1-W26	E001–E040	40	1.54	4	Yes	–
L1	S1A.	W27-W33	E0040–E050	10	1.43	5	Yes	Training
	S1B.	W27-W33	E0042–E052	10	1.43	5	Yes	Training
L2	S2A.	W27-W33	E0026–E033	7	1.00	7	Yes	Training
	S2B.	W27-W33	E0020–E026	6	0.86	8	Yes	Training
L3	S3A.	W27-W33	E0026–E033	7	1.00	7	Yes	Testing
	S3B.	W27-W33	E015–E021	6	0.86	8	Yes	Testing
L4	S4A.	W27-W33	E080–E099	19	2.71	3	Yes	Testing
	S4B.	W27-W33	E040–E050	10	1.43	4	Yes	Testing

It can be observed that the ESDD measurements for some insulator strings were repeated more than twice per week, in accordance with the procedures specified in IEC 60507 [54]. These insulator strings are situated in locations with significantly higher pollution levels than the other sites. Consequently, additional measurements were performed to improve the accuracy and consistency of the ESDD assessment and to capture the variability of contamination accumulation in these highly polluted environments.

During model training, each experiment was independently repeated multiple times to ensure robustness and reproducibility. The reported results represent the mean performance across all runs, while the variability is quantified as mean ± standard deviation (SD). Detailed results for all repetitions are provided in the **Supplementary Materials**.

### Pre-optimization model performance

(a) Weeks 1–26

**Fig 6:**
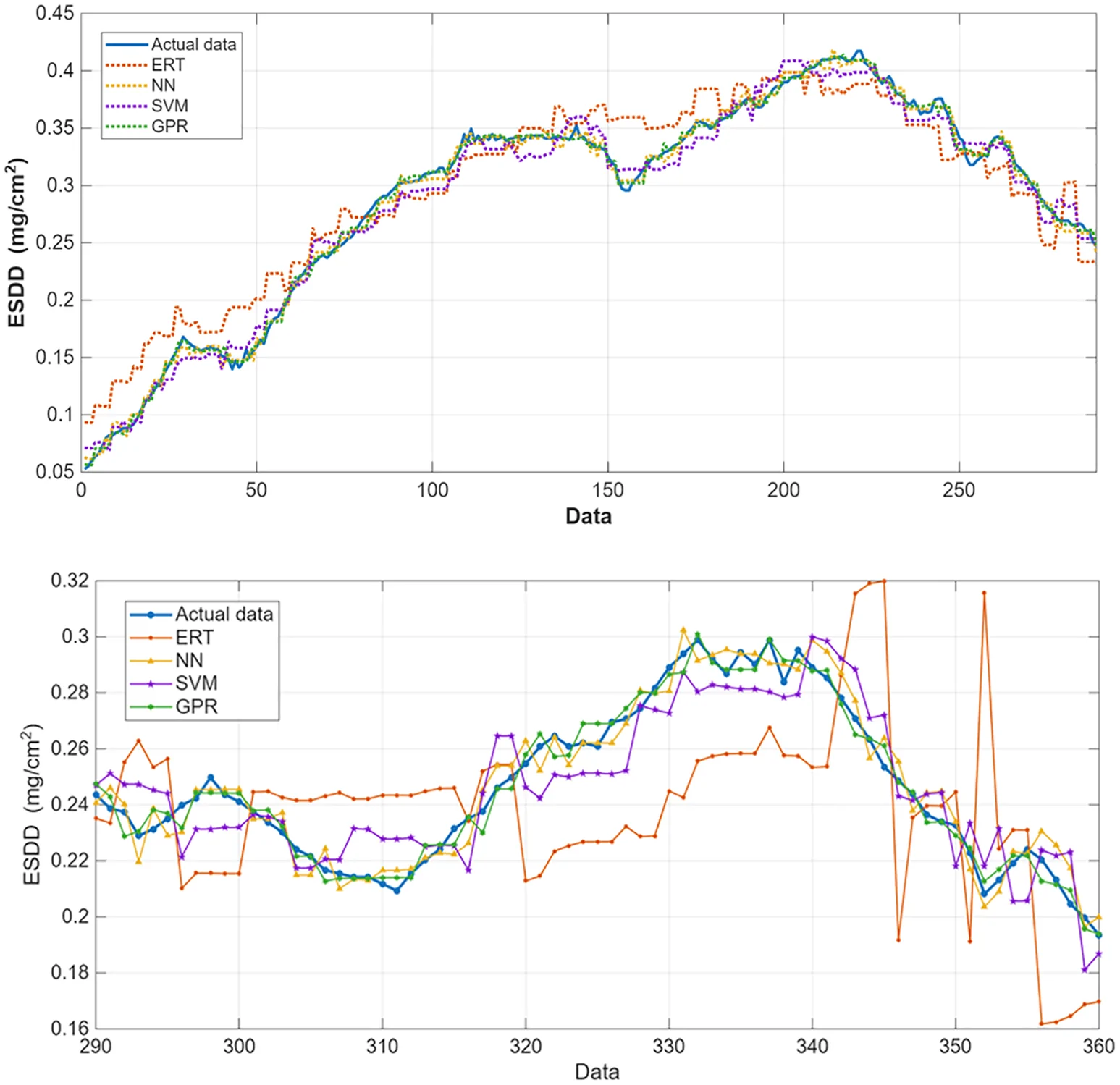
Predicted ESDD Results without optimization in scenario 1 (a) training, (b) testing.

**Fig 7:**
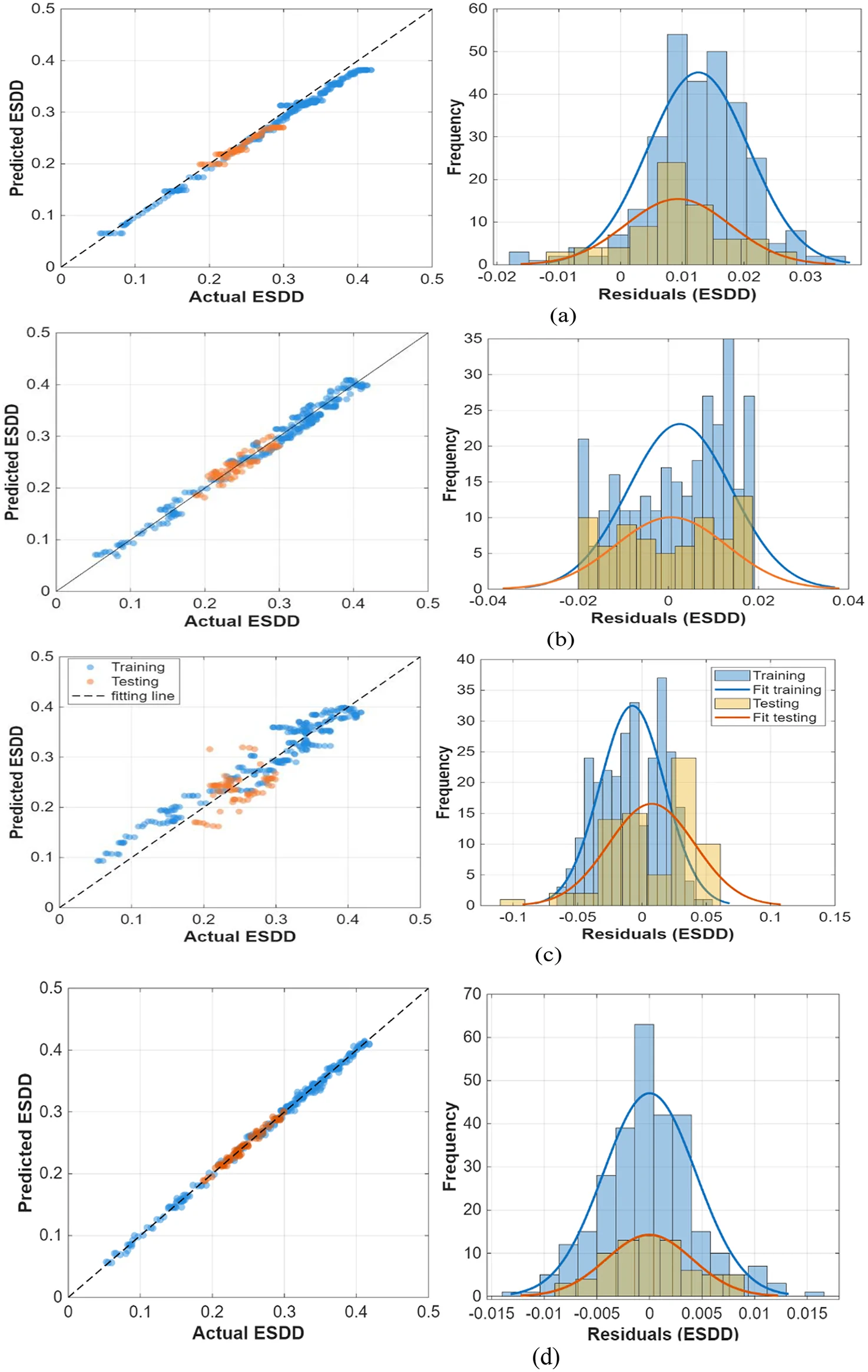
Regression results and error histogram of pre-optimized models in scenario 1: (a) SVM, (b) NN,(c) ERT, (d) GPR.

To explore the effects of optimisation on model performance, the suggested models were trained before their implementation. Fig 6 and Fig 7 display the results of training and testing the ESDD prediction models in scenario 1, as an example, including a comparison of the predicted values with the actual values, the differences between them, and a graph illustrating the relationship between the predicted and actual responses. Table 5 also includes the mean evaluation metric outcomes, prediction speed, and training time for the proposed models without optimization for the four scenarios. The results shown in Figs 6 **and** 7 and Table 8 show that the proposed models perform well even when optimization is not applied to their hyperparameters, especially with scenario 1 splitting. Among the proposed models, GPR performs the best in both training and testing. With an RMSE of 12.52 × 10 ⁻ ^3^ and 6.124 × 10 ⁻ ^3^ for training and testing, respectively; 165.91 × 10 ⁻ ⁶ and 137.501 × 10 ⁻ ⁶ of the MSE for training and testing, respectively; an MAE of 9.144 × 10 ⁻ ^3^ for training and 5.2814 × 10 ⁻ ^3^ for testing; an R^2^ of 0.9788 for training and 0.9718 for testing; and 3.7% and 5.2% of MAPE for training and testing, respectively, the GPR exhibits a modest advantage over all models. In contrast, the SVM outperforms the GPR in terms of prediction speed and model size by approximately 52 kB/s, compared to 23 kB/s for the GPR, and 8 kB compared to 17 kB for the GPR. Notably, the NN achieved the fastest model with a training time of 3.187 seconds. Table 5 reports that model performance before optimization varies across scenarios, with **Scenario 2** (Combined: Leave-One-Location + Time-Blocked) yielding the best overall accuracy, followed by Scenario 3 and Scenario 4.

**Table 8:** Performance metrics results of the proposed models before optimization for all scenarios.

Model	RMSE ×10^−3^	MSE ×10^−6^	MAE ×10^−3^	R^2^	MAPE%	Speed (kB/s)	Training time (s)	Model size kB
Training results for scenario 1								
**SVM**	13.43	180.48	11.274	0.9756	4.7	**52**	93.159	**8**
**NN**	15.29	233.89	13.512	0.9684	5.1	9	**3.187**	59
**ERT**	25.66	658.48	21.013	0.9111	8.8	5.2	109.99	19
**GPR**	12.52	165.91	9.144	0.9788	3.7	23	21.63	17
**Testing results for scenario 1**								
**SVM**	11.87	140.97	10.469	0.9707	6.2	–	–	–
**NN**	14.55	211.82	12.914	0.9610	5.6	–	–	–
**ERT**	27.97	782.52	23.566	0.8930	11.1	–	–	–
**GPR**	6.124	137.501	5.2814	0.9718	5.2	–	–	–
**Training results for scenario 2**								
**SVM**	**10.634**	**114.77**	**5.762**	**0.9825**	**5.23**	**21**	**53.62**	46
**NN**	**12.557**	**206.14**	**11.471**	**0.9775**	**5.61**	**19**	**13.57**	13
**ERT**	**15.644**	**587.95**	**12.873**	**0.9371**	**7.93**	**33**	**39.24**	23
**GPR**	**10.375**	**107.43**	**5.198**	**0.9842**	**4.13**	**24**	**21.18**	21
**Testing results for scenario 2**								
**SVM**	**11.884**	**109.51**	**8.662**	**0.9784**	**4.02**	–	–	–
**NN**	**12.073**	**201.54**	**10.713**	**0.9638**	**3.68**	–	–	–
**ERT**	**13.445**	**589.46**	**12.223**	**0.9364**	**6.92**	–	–	–
**GPR**	**10.884**	**99.722**	**5.633**	**0.9802**	**3.69**	–	–	–
Training results for scenario 3								
SVM	11.23	146.5	9.14	0.9779	6.1	24	71.6	78
NN	13.67	234.91	13.35	0.9632	6.4	15	21.74	35
ERT	22.38	699.12	14.76	0.9260	9.2	26	67.3	29
GPR	12.34	146.23	7.75	0.9793	4.5	11	43.8	25
Testing results for scenario 3								
SVM	12.37	136.13	12.23	0.9747	4.13	–	–	–
NN	14.94	248.46	15.13	0.9621	4.45	–	–	–
ERT	25.63	686.65	19.28	0.9099	7.21	–	–	–
GPR	14.03	181.76	7.71	0.9760	3.87	–	–	–
Training results for scenario 4								
**SVM**	11.56	178.78	10.42	0.9761	6.2	25.3	61.5	12
**NN**	13.12	247.57	13.68	0.9662	6.4	26.9	19.9	37
**ERT**	27.56	714.34	18.89	0.9136	10.2	28.5	68.63	27
**GPR**	10.41	160.13	7.83	0.9791	4.5	19	27.4	31
Testing results for scenario 4								
**SVM**	13.37	184.87	12.44	0.9736	4.4	–	–	–
**NN**	15.26	255.11	15.19	0.9601	4.5	–	–	–
**ERT**	29.44	726.78	19.32	0.9086	7.6	–	–	–
**GPR**	14.54	189.31	7.96	0.9749	4.9	–	–	–

### Optimized model results

#### Optimized SVM model.

**Fig 8:**
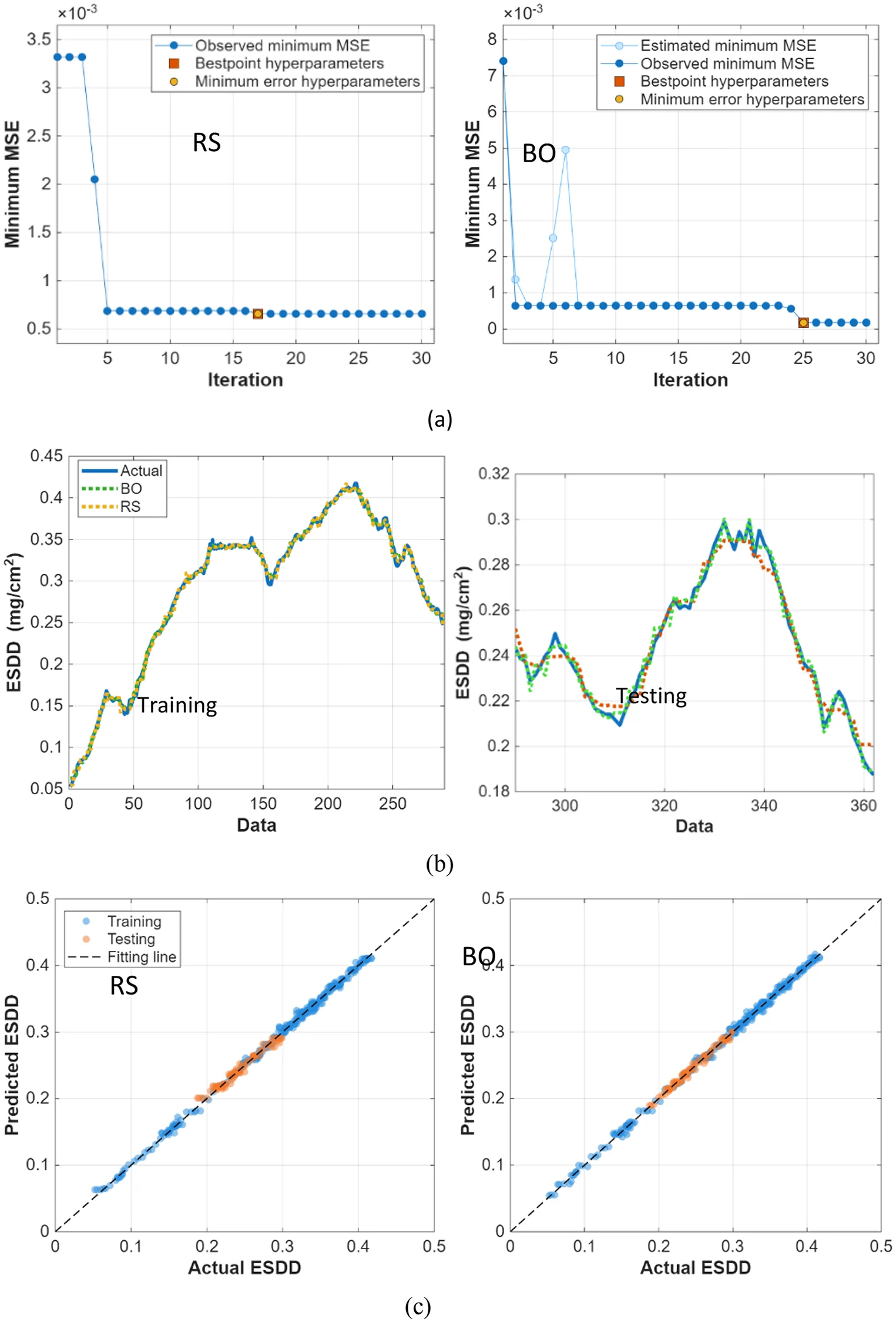
Scenario 1 (a) MSE, (b) series time plot, and (c) Regression results of Optimized SVM.

To provide accurate ESDD predictions, the SVM model considers how the input variables interact and connect. The parameters of the model, such as “box constraint, epsilon value, kernel type, and whether the data is standardized”, all play an important role in determining the prediction accuracy of the model performance. In tuning SVM hyperparameters, Bayesian Optimization (BO) proved better than Random Search (RS), as illustrated in Fig 8. The results in Fig 8 and the evaluation metrics in Table 9 show that the BO-optimized SVM created a simpler, more regularized model with effective learning by using a Gaussian kernel with a small box constraint (157.83) and wider epsilon (6.2E-05). Table 9 offers a revised comparison of performance metrics for SVM models optimized through Random Search (RS) and Bayesian Optimization (BO) in both training and testing phases. During the training phase, using scenario 1 as an example, the RS-based model shows slightly higher RMSE (7.375 × 10 ^-^ ^3^) and MSE (5.439 × 10 ^-^ ^5^) compared to BO (RMSE = 5.831 × 10 ^-^ ^3^; MSE = 3.435 × 10 ⁻ ^5^), indicating that BO provides a better fit to the training data. Similarly, the BO-optimized model achieves a lower MAE of 4.315 × 10 ^-^ ^3^ compared to 4.493 × 10 ^-^ ^3^ RS. Although the R^2^ values are very close (0.9955 for RS and 0.9956 for BO), BO attains a slightly lower MAPE (2.0% vs. 2.3%). However, this improvement comes with a trade-off in computational resources: BO requires significantly more training time (30.39 s vs. 8.026 s) and results in a larger model size (151 kB vs. 16 kB), though the prediction speed remains consistent at 20 kB/s for both. In the testing phase, BO demonstrates superior generalization capability across all primary metrics. The BO-optimized model records lower RMSE (3.211 × 10 ⁻ ^3^ vs. 3.787 × 10 ^-^ ^3^), MSE (1.173 × 10 ⁻ ⁵ vs. 1.443 × 10 ⁻ ⁵), and MAE (3.204 × 10 ^-^ ^3^ vs. 5.568 × 10 ^-^ ^3^) than its RS counterpart. Moreover, BO slightly surpasses RS in terms of R^2^ (0.99857 vs. 0.99804), confirming its robustness and higher predictive accuracy on unseen data. The most notable improvement is seen in MAPE, where BO achieves 1.1%, outperforming RS’s 1.3%. While BO increases training costs and model complexity, it consistently yields better performance metrics, especially in testing, indicating its effectiveness in enhancing model generalization for SVMs. Moreover, regression plots and absolute error analysis confirmed BO’s superior generalization and forecasting accuracy. Across all scenarios, Scenario 2 demonstrates the best overall performance with the lowest RMSE and highest 𝑅^2^, followed by Scenario 3 and Scenario 4, while Scenario 1 shows comparatively lower accuracy. Bayesian Optimization (BO) generally improves training performance, whereas Random Search (RS) often provides slightly better or comparable results in testing, indicating more stable generalization.

**Table 9:** Performance metrics for optimized SVM models with RS and BO across all scenarios.

Model	Opt.	RMSE ×10^−3^	MSE ×10^−5^	MAE ×10^−3^	R^2^	MAPE%	Speed kB/s	Time s	Model size kB
Scenario 1									
SVM Training	RS	7.37	5.439	4.493	0.9955	2.3	20	8.026	16
	BO	5.83	3.435	4.315	0.9956	2	20	30.39	151
SVM Testing	RS	8.78	1.434	5.568	0.9980	1.3	–	–	–
	BO	4.21	1.173	3.204	0.9985	1.1	–	–	–
**Scenario 2**									
SVM Training	RS	**5.57**	**2.899**	**3.312**	**0.9973**	**1.8**	**21**	**10.6**	**14**
	BO	**2.47**	**2.938**	**3.198**	**0.9881**	**1.6**	**22**	**17.15**	**81**
SVM Testing	RS	**2.67**	**0.877**	**3.565**	**0.9984**	**0.59**	–	–	–
	BO	**2.86**	**0.794**	**2.086**	**0.9990**	**0.487**	–	–	–
Scenario 3									
SVM Training	RS	6.23	3.963	4.256	0.9960	1.8	32	16.4	22
	BO	3.65	3.224	3.982	0.9966	1.7	17	24.47	77
SVM Testing	RS	3.12	1.356	4.872	0.9983	0.68	–	–	–
	BO	3.04	1.029	2.146	0.9986	0.603	–	–	–
Scenario 4									
SVM Training	RS	6.37	4.97	4.311	0.9958	2.1	28	12.73	12.5
	BO	3.83	3.36	4.146	0.9963	1.9	21	22.98	46
SVM Testing	RS	3.32	1.39	5.268	0.9982	0.76	–	–	–
	BO	3.08	1.038	2.902	0.9985	0.623	–	–	–

#### Optimized NN model.

**Fig 9:**
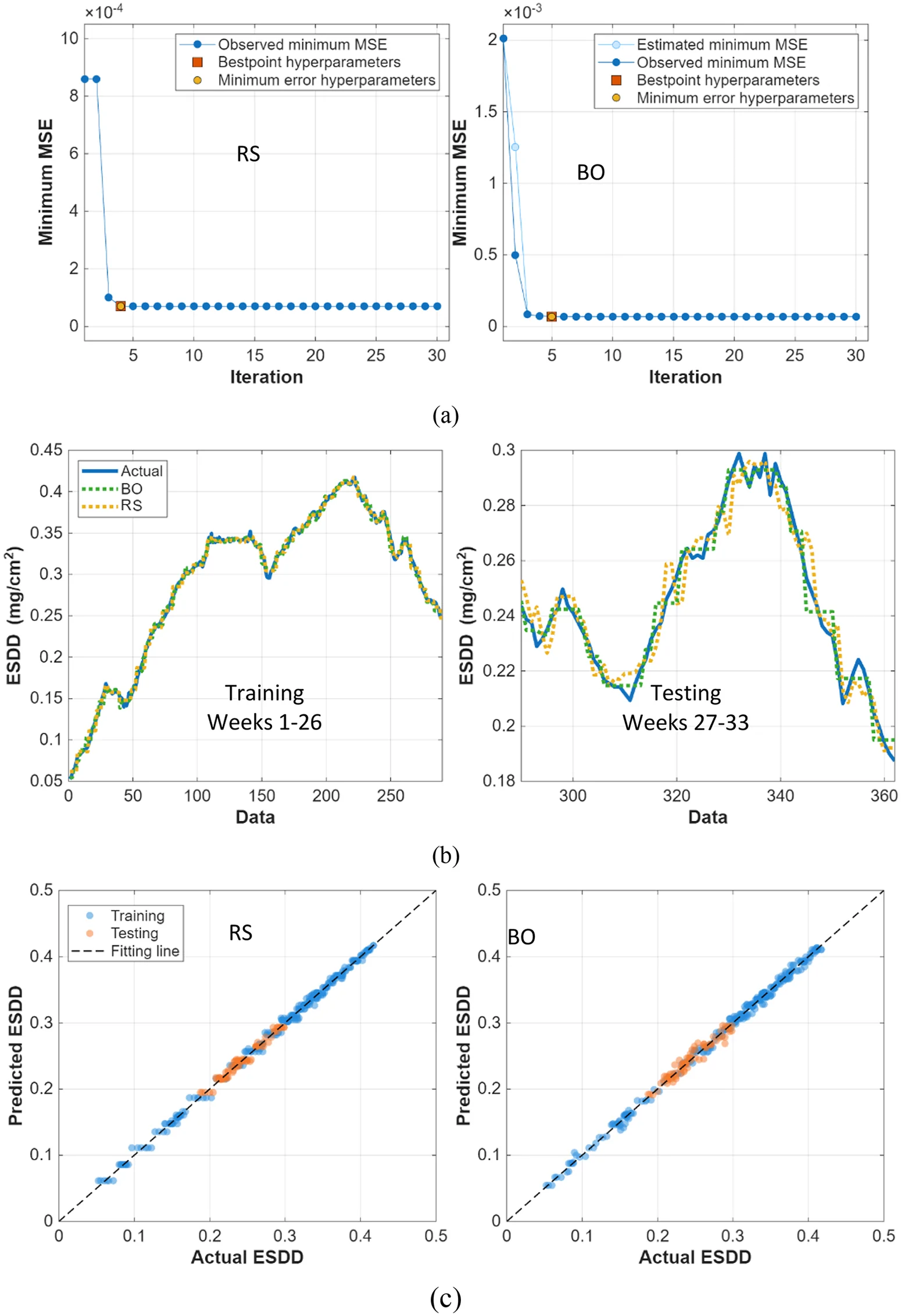
(a) MSE, (b) series time plot, and (c) Regression results of Optimized NN.

NN has proven effective in forecasting ESDD by capturing intricate patterns within the data. BO and RS were applied to fine-tune key NN hyperparameters, including the number of hidden layers, neurons per layer, activation functions (ReLU, Tanh, Sigmoid), and regularization strength (lambda). Among the tested activation functions, ReLU delivered the best performance due to its efficiency in learning complex patterns, avoiding vanishing gradients, and reducing computational burden. This contributed to a lower required regularization strength (λ = 1.932 for BO and 0.089 for RS), indicating minimal risk of overfitting. The optimized model results are shown in Fig 9 and Table 10. The results show that the optimized model achieved a low index and high regression compared to NN without optimization. Performance analysis revealed that the optimal hyperparameter set was reached early in the training process for both BO and RS, with the best configuration and minimum error occurring at iterations 4 and 5, respectively. The performance comparison between neural network (NN) models optimized using Random Search (RS) and Bayesian Optimization (BO) for scenario 1 is presented in Table 10, highlighting key metrics for both training and testing phases. During training, BO demonstrates slightly superior performance with marginal improvements in RMSE (8.166 × 10 ⁻ ³ vs. 8.317 × 10 ⁻ ³), MSE (6.668 × 10 ⁻ ⁵ vs. 6.917 × 10 ⁻ ⁵), MAE (6.127 × 10 ⁻ ³ vs. 6.149 × 10⁻ ³), and R^2^ (0.9909 vs. 0.9906) compared to RS. Additionally, BO achieves a lower MAPE% (2.6%) than RS (2.7%), and offers a more efficient model with a 40.7% reduction in training time (7.124 s vs. 12.02 s) and a 54.5% smaller model size (10 kB vs. 22 kB), though RS maintains a higher prediction speed (27 kB/s vs. 22 kB/s). However, in the testing phase, RS significantly outperforms BO across all evaluation metrics, achieving lower RMSE (4.656 × 10 ⁻ ³ vs. 5.362 × 10 ⁻ ³), MSE (2.168 × 10 ⁻ ⁵ vs. 2.876 × 10 ⁻ ⁵), MAE (3.495 × 10 ⁻ ³ vs. 4.153 × 10 ⁻ ³), and MAPE% (1.6% vs. 1.8%), along with a higher R^2^ value (0.99703 vs. 0.99606). These results indicate that while BO provides faster and more compact models during training, RS yields superior generalization performance on unseen data, making it more favourable for predictive accuracy. Across all scenarios, the NN model shows strong predictive performance, with Scenario 2 achieving the best results in terms of lowest RMSE and highest 𝑅^2^, followed by scenarios 3 and 4, while Scenario 1 performs comparatively weaker.

**Table 10:** Performance metrics for optimized NN models with RS and BO across all scenarios.

Model	Opt.	RMSE ×10^−3^	MSE ×10^−5^	MAE ×10^−3^	R^2^	MAPE%	Speed kB/s	Training time s	Model size kB
**Scenario 1**									
**NN** **Training**	RS	8.317	6.917	6.149	0.9906	2.7	27	12.02	22
	BO	8.166	6.668	6.127	0.9909	2.6	22	7.124	10
**NN** **Testing**	RS	4.656	2.168	3.495	0.9970	1.6	–	–	–
	BO	5.362	2.876	4.153	0.9960	1.8	–	–	–
**Scenario 2**									
NN Training	RS	**5.725**	**4.828**	**3.648**	**0.9924**	**2.2**	**27**	**11.24**	**20**
	BO	**3.864**	**4.172**	**3.053**	**0.9937**	**1.7**	**24**	**12.38**	**18**
NN Testing	RS	**2.331**	**1.877**	**3.264**	**0.9986**	**0.9**	–	–	–
	BO	**2.965**	**1.216**	**3.518**	**0.9978**	**1.08**	–	–	–
**Scenario 3**									
NN Training	RS	6.557	5.895	4.252	0.9912	2.4	24	15.3	9.9
	BO	4.987	4.405	4.032	0.9928	2.3	16	9.39	12.3
NN Testing	RS	2.366	2.027	3.325	0.9984	1.2	–	–	–
	BO	3.019	2.526	4.100	0.9976	1.3	–	–	–
**Scenario 4**									
**NN** **Training**	RS	6.687	5.944	4.491	0.9909	2.6	23	10.43	4.2
	BO	5.773	4.686	4.172	0.9912	2.4	10	6.82	6.8
**SVM** **Testing**	RS	2.566	2.145	3.459	0.9980	1.5	–	–	–
	BO	3.623	2.761	4.103	0.9970	1.6	–	–	–

#### Optimized ERT model.

**Fig 10:**
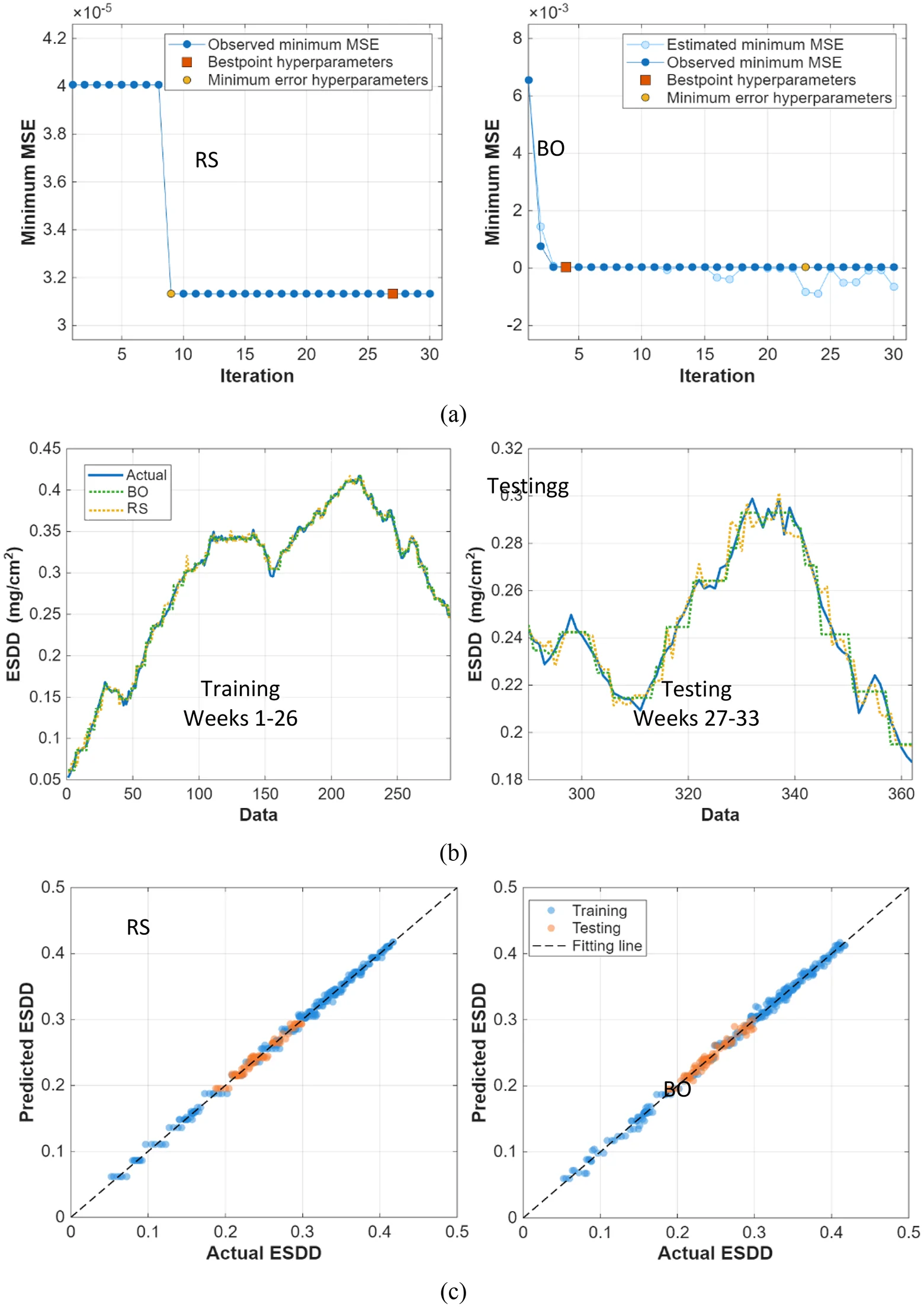
Scenario 1(a) MSE, (b) series time plot, and (c) Regression results of Optimized ERT.

Fig 10 shows the results of the optimized ERT Model in scenario 1. For the ERT model, BO optimizes several key parameters, including the ensemble method, minimum leaf size, number of trees, number of features to sample, and learning rate. BO prefers LSBoost because of its lower bias and its ability to model complex patterns, even though it is more susceptible to overfitting. With LSBoost, the model achieved in scenario 1 a high prediction speed (22 kB/s) and reduced training time (7.124 s), surpassing RS in these aspects. As shown in Fig 10(a), the minimum mean squared error (MSE) plot for BO showed stabilizing performance early on, fluctuating in later iterations, which reflects the model’s learning dynamics and noise sensitivity. Key hyperparameter milestones were achieved at iterations 8 (lowest error) and 8 (best configuration). The regression plot (Fig 10(C)) revealed a strong correlation between predicted and actual ESDD outputs. RS, on the other hand, explores the hyperparameter space by randomly sampling combinations, which can uncover effective configurations without needing a defined grid. For RS, optimal values included a minimum leaf size of 43. RS chose bagging as the ensemble method, which, while more stable and less prone to overfitting, may not capture complex data relationships as effectively as LSBoost. Table 11 shows that BO improved ERT model performance over RS, for example, in scenario 1, BO optimization reduced RMSE from 5.196 × 10 ⁻ ³ to 4.656 × 10 ⁻ ³ and MSE from 2.700 × 10 ⁻ ⁵ to 2.168 × 10 ⁻ ⁵ in the test dataset compared to the RS method. BO also achieved MAE (3.495 × 10 ⁻ ³) and R² (0.9970), decreasing model size (10 kB). Overall, while BO enabled faster training and better model complexity handling through LSBoost, RS produced competitive results using Bagging, demonstrating good generalization and lower sensitivity to hyperparameter tuning. The choice between BO and RS depends on the trade-off between model complexity, accuracy, and computational efficiency. The ERT model achieves its best performance in scenario 2, particularly with BO during testing, where the RMSE, MSE, and MAE reach their lowest values of 3.695 × 10 ⁻ ³, 0.972 × 10 ⁻ ^5^, and 2.594 × 10 ⁻ ³, respectively, with a high 𝑅^2^ = 0.9978 and MAPE of 1.4%. In comparison, scenario 3 also performs well (RMSE = 3.993 × 10 ⁻ ³, 𝑅^2^ = 0.9965), but remains slightly inferior. Overall, scenario 2 (Leave-One-Location) with BO optimization provides the most accurate and reliable results for the ERT model, outperforming all other scenarios.

**Table 11:** Performance metrics for optimized ERT models with RS and BO across all scenarios.

Model	Opt.	RMSE ×10^−3^	MSE ×10^−5^	MAE ×10^−3^	R^2^	MAPE%	Speed kB/s	Time s	Model size kB
**Scenario 1**									
**ERT** **Training**	RS	8.820	7.779	6.387	0.9894	2.7	14	4.561	10
	BO	8.317	6.917	6.149	0.9906	2.7	23	7.236	22
**ERT** **Testing**	RS	5.196	2.700	4.025	0.9963	1.8	–	–	–
	BO	4.656	2.168	3.495	0.9970	1.7	–	–	–
**Scenario 2**									
**ERT** **Training**	RS	**4.843**	**2.973**	**4.238**	**0.9912**	**2.1**	23	11.03	21
	BO	**4.396**	**2.845**	**4.319**	**0.9918**	**2.3**	**32**	8.28	11
**ERT** **Testing**	RS	**4.365**	**2.034**	**3.251**	**0.9972**	**1.5**	–	–	–
	BO	**3.695**	**0.972**	**2.594**	**0.9978**	**1.4**	–	–	–
**Scenario 3**									
**ERT** **Training**	RS	7.116	6.814	4.591	0.9898	2.2	13	17.2	20
	BO	7.573	6.012	5.882	0.9915	2.8	28	7.4	12
**ERT** **Testing**	RS	4.925	2.281	3.921	0.9961	1.6	–	–	–
	BO	3.993	1.695	2.890	0.9965	1.4	–	–	–
**Scenario 4**									
**ERT** **Training**	RS	7.280	7.191	5.873	0.9897	2.5	14	17.9	23
	BO	7.713	6.271	5.941	0.9913	2.6	31	5.6	9.6
**ERT** **Testing**	RS	5.104	2.404	3.952	0.9956	1.9	–	–	–
	BO	4.065	1.868	3.594	0.9960	1.6	–	–	–

#### Optimized GPR model.

**Fig 11:**
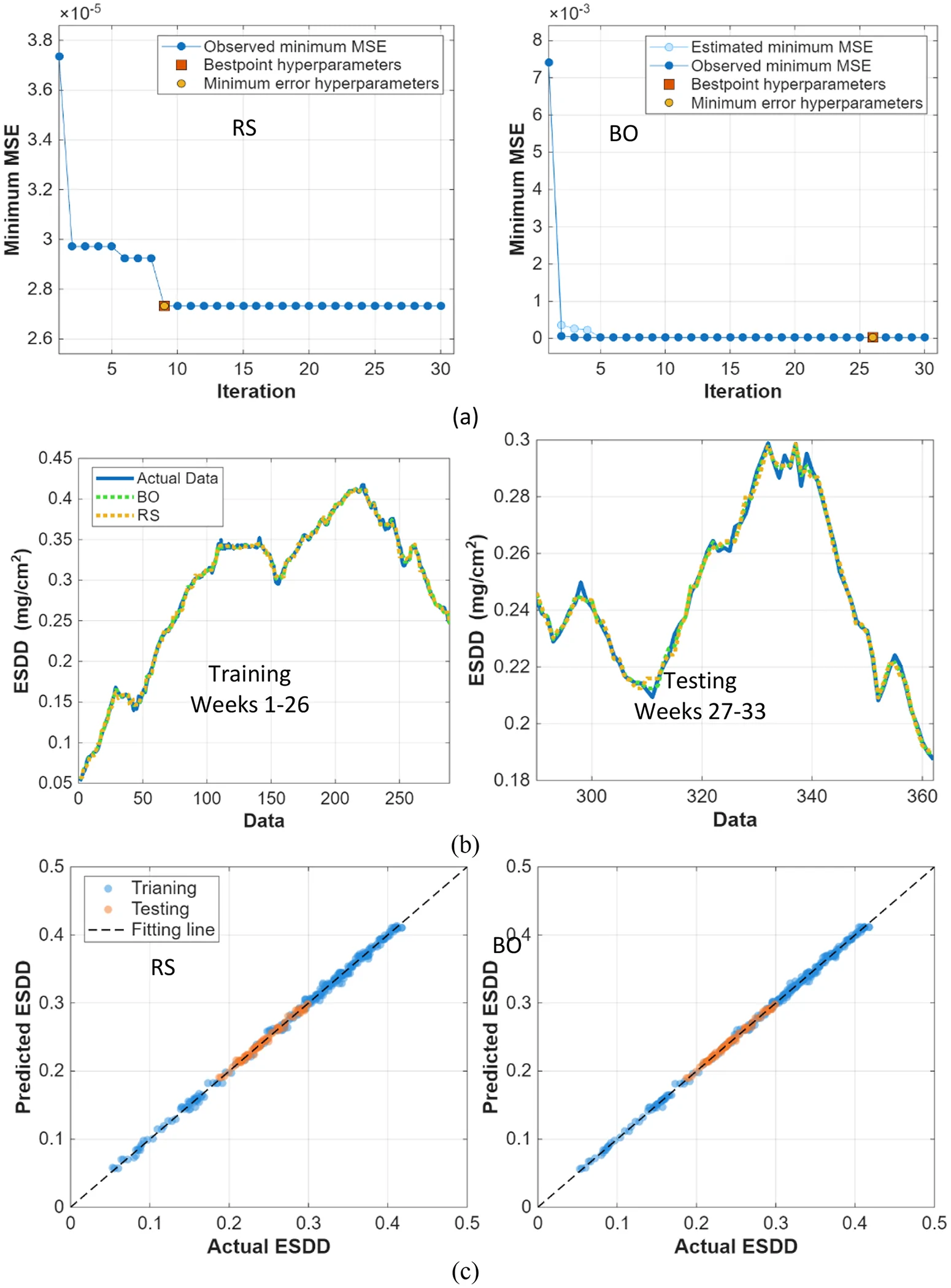
Scenario 1 (a) MSE, (b) series time plot, and (c) Regression results of Optimized GPR.

GPR was utilized for ESDD prediction by learning from the meteorological and electrical properties output data. The model proved effective in identifying data trends and providing forecasts at fine time intervals, which is valuable for energy management and grid operations. To enhance GPR performance, hyperparameter tuning was conducted using BO and RS. The BO-tuned model performed well by choosing the best values for sigma (1.693E-04), kernel scale (15.48), a Linear basis function, and the nonisotropic Matern 3/2 kernel. These settings resulted in reduced noise, smoother predictions, and better adaptability to input variations. As shown in Fig 11(a), the convergence plot showed optimal results at iteration 26, and the best hyperparameter configuration achieved the lowest error at the same point, indicating effective optimization. In contrast, the RS-optimized GPR used a higher sigma (0.00172) and kernel scale (3.8635), suggesting a more generalized but potentially less sensitive model. While RS also yielded excellent results, as presented in Fig 11, the BO-based model demonstrated superior accuracy, lower RMSE, faster predictions, and slightly quicker training. Table 12 presents the performance metrics of optimized GPR models using Random Search (RS) and Bayesian Optimization (BO). BO consistently outperforms RS in both training and testing phases. During testing, the BO-optimized model, when trained using scenario1 splitting as an example, achieved the lowest RMSE (2.699 × 10 ⁻ ³), MSE (0.728 × 10 ⁻ ⁵), and MAE (1.891 × 10 ⁻ ³), along with the highest R^2^ value (0.9983) and lowest MAPE (0.8%). In training, BO also showed better performance with lower RMSE, MSE, MAE, and faster training time (2.879 s) compared to RS (4.682 s). Additionally, the BO model had a smaller size (50 kB) and comparable prediction speed (14 kB/s) relative to RS (17 kB/s). The GPR model achieves its best overall performance in scenario 2, particularly with BO during testing, where it records the lowest RMSE (1.082 × 10 ⁻ ³), and highest 𝑅^2^ (0.9991). Scenarios 3 and 4 also show strong performance, but slightly inferior compared to scenario 2. Overall, Scenario 2 with BO optimization provides the most accurate and reliable results for the GPR model.

**Table 12:** Performance metrics results for optimized GRP models with RS and BO across all scenarios.

Model	Opt.	RMSE ×10^−3^	MSE ×10^−5^	MAE ×10^−3^	R^2^	MAPE%	Speed	Time s	Model size
**Scenario 1**									
**GPR Training**	RS	5.748	3.304	4.493	0.9955	1.9	17	4.682	55
	BO	5.225	2.729	4.059	0.9963	1.7	14	2.879	50
**GPR Testing**	RS	3.671	1.347	2.786	0.9981	1.2	–	–	–
	BO	2.699	0.728	1.891	0.9983	0.8	–	–	–
**Scenario 2**									
**GPR Training**	RS	**2.325**	**2.356**	**2.245**	**0.9982**	**1.2**	**27**	**3.21**	**24**
	BO	**1.624**	**1.179**	**1.926**	**0.9983**	**1.1**	**25**	**3.81**	**31**
**GPR Testing**	RS	**2.275**	**1.147**	**1.851**	**0.9987**	**0.92**	–	–	–
	BO	**1.082**	**0.598**	**1.032**	**0.9991**	**0.51**	–	–	–
**Scenario 3**									
**GPR Training**	**RS**	**4.681**	**2.919**	**4.212**	**0.9971**	**1.3**	**20**	**5.32**	**21**
	**BO**	**4.091**	**2.023**	**2.899**	**0.9982**	**1.2**	**17**	**4.36**	**19**
**GPR Testing**	**RS**	**2.464**	**1.156**	**2.043**	**0.9985**	**0.98**	**–**	**–**	**–**
	**BO**	**1.162**	**0.623**	**1.321**	**0.9988**	**0.57**	**–**	**–**	**–**
**Scenario 4**									
**GPR Training**	RS	4.847	3.005	4.251	0.9962	1.6	20	4.97	25
	BO	4.252	2.126	3.795	0.9978	1.4	18	4.84	18
**GPR Testing**	RS	2.762	1.276	2.088	0.9983	1.1	–	–	–
	BO	1.239	0.688	1.494	0.9986	0.6	–	–	–

**Fig 12:**
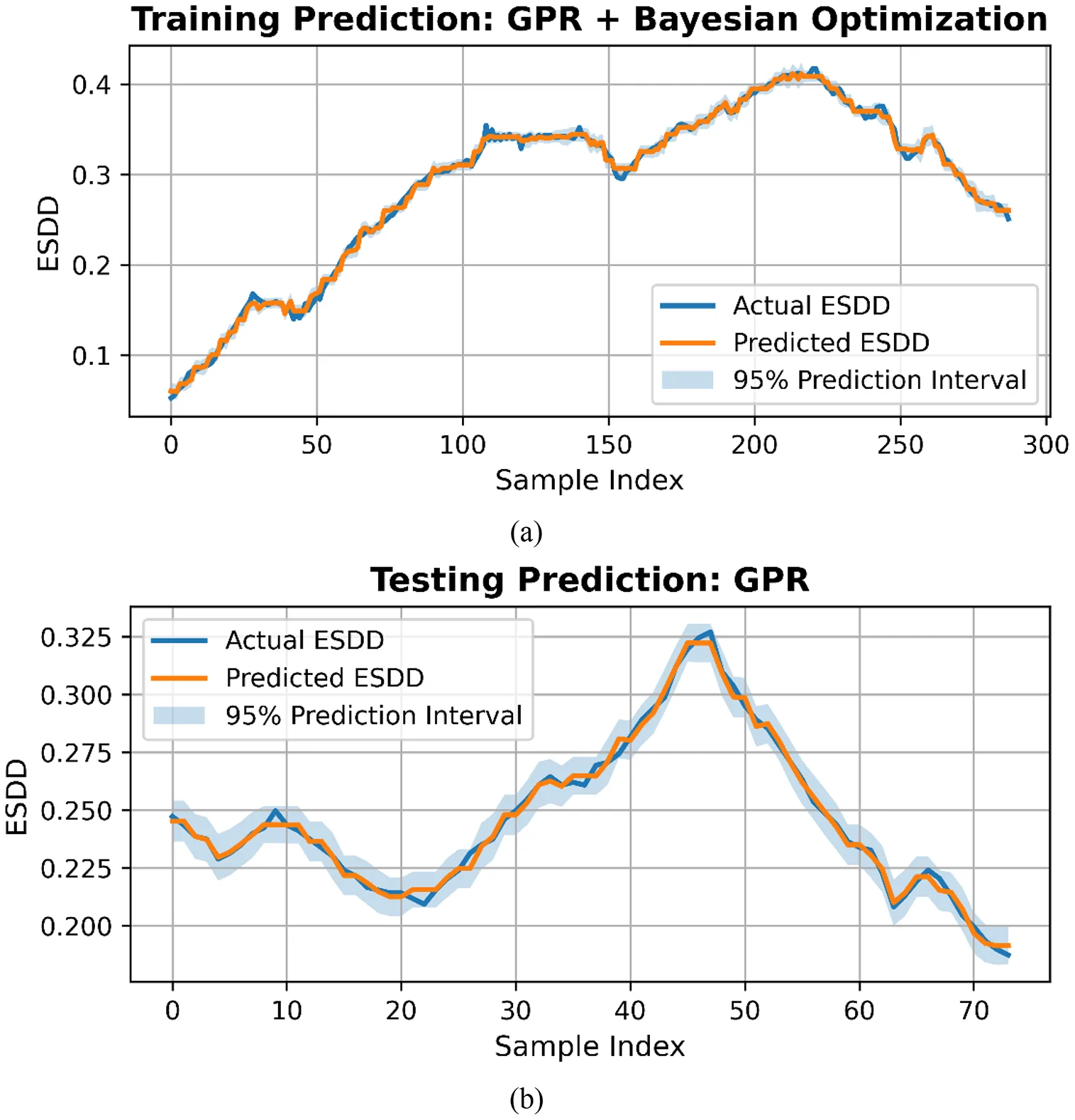
GPR-BO model with uncertainty intervals: (a) training, (b) testing.

Overall, GPR with BO produced the best forecasting performance among all tested models and optimizers, owing to its ability to effectively explore the hyperparameter space and fine-tune model behavior. Fig 12 illustrates the performance of the GPR-BO model with associated 95% uncertainty intervals with scenario 1, showing close agreement between predicted and actual ESDD values. The narrow and well-contained intervals indicate that the model produces stable predictions with well-calibrated uncertainty estimates. GPR achieved the most accurate model, followed by SVM, NN, and ERT. The only notable limitation of GPR is its computational cost, as training times are approximately twice as long as those of some other models. Across most models, particularly SVM, NN, and GPR, the Bayesian Optimization (BO) approach yielded better results than Random Search (RS), highlighting the impact of efficient hyperparameter tuning. Even within the ERT model, BO delivered faster predictions and shorter training durations, although with a slight trade-off in accuracy. The effectiveness of hyperparameter tuning is evident in the significant error reductions achieved in this study. Finally, the findings highlight that the combination of an appropriate model (especially GPR), optimal scenario (scenario 2), and balanced optimization strategy (BO for training and RS for testing) yields the most accurate and efficient predictive performance for the studied application followed by. Scenario 4 is only a supplementary benchmark for comparison purposes.

### Interpretability analysis

**Fig 13:**
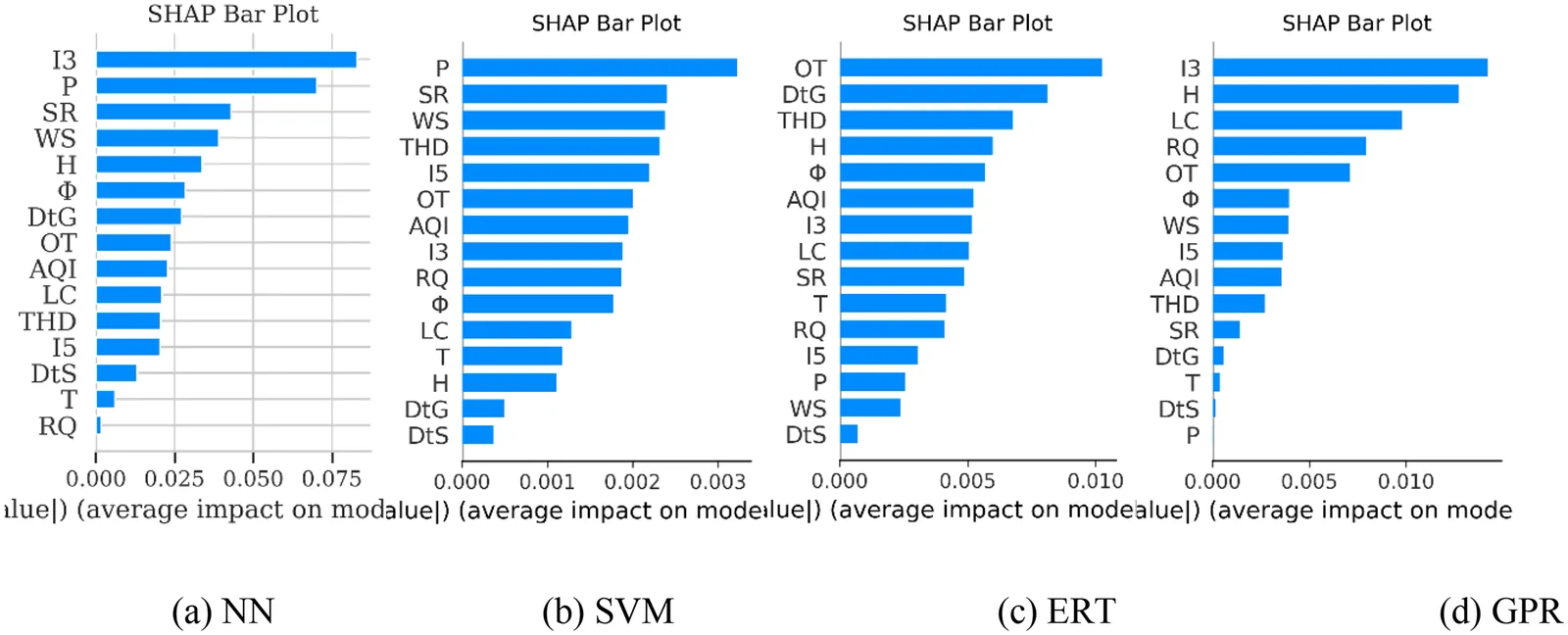
SHAP results for all 15 features of the proposed methods.

To investigate the relationship between the selected features and ESDD, and to improve the interpretability of advanced machine learning models, the SHAP (SHapley Additive exPlanations) method was applied. SHAP provides a unified framework for interpreting model outputs by estimating feature contributions based on Shapley values from cooperative game theory. This approach enables both local and global explanations, offering insight into how input variables influence model predictions. However, it is important to note that SHAP explains model behavior rather than establishing direct physical causality. Given the presence of strong multicollinearity among several predictors (with correlation coefficients approaching 0.99), SHAP-based feature attributions should be interpreted with caution. In such cases, importance may be distributed among correlated variables, making it difficult to isolate independent contributions. Therefore, the reported feature importance reflects model-dependent attribution within the given feature space rather than definitive identification of physical drivers of ESDD. SHAP values were computed using model-appropriate explainers. For the ERT model, TreeExplainer was used because it is designed for tree-based ensemble models. For the SVM and GPR models, KernelExplainer was applied as a model-agnostic SHAP approach. For the NN model, DeepExplainer was used to estimate feature contributions in the neural network structure. The background data used for SHAP estimation were selected from the training set within each cross-validation fold to avoid data leakage. Fig 13 presents a comparison of feature importance across four machine learning models (NN, SVM, ETR, and GPR), using SHAP-based visualisations. The observed variation in feature rankings across models highlights differences in how each algorithm responds to feature interactions, nonlinear relationships, and data structure. The variation in feature importance across different models, as illustrated in Fig 13(a)-(d), highlights the diverse ways in which machine learning algorithms interpret and prioritize input variables when predicting ESDD. In the NN model, features such as ‘I3’, ‘P’, and ‘SR’ are identified as highly influential within the model, reflecting its ability to capture complex nonlinear dependencies and interactions. The SVM model assigns relatively higher importance to features such as ‘P’, ‘SR’, and ‘WS’, consistent with their role in defining the separation boundary in the transformed feature space. In the ETR model, features including ‘OT’, ‘DtG’, ‘THD’, and ‘H’ are frequently selected as important, as they contribute to variance reduction across randomized tree splits. Similarly, the GPR model attributes higher importance to features such as ‘I3’, ‘H’, ‘LC’, and ‘RQ’, reflecting their influence within the probabilistic framework and kernel-based structure of the model. It is noteworthy that feature rankings differ across models, which underscores the sensitivity of attribution methods to both model structure and correlated inputs. While some variables (e.g., ‘I3’, ‘H’, and ‘OT’) appear consistently among the more influential features, their relative importance varies and should be interpreted as conditional on the model rather than as independent physical effects. To enhance the robustness of the interpretation, SHAP results were considered alongside permutation feature importance analysis. Broad agreement was observed for several key features, although some discrepancies were noted, particularly among highly correlated variables. This further highlights the need for cautious interpretation when attributing importance in the presence of multicollinearity.

**Fig 14:**
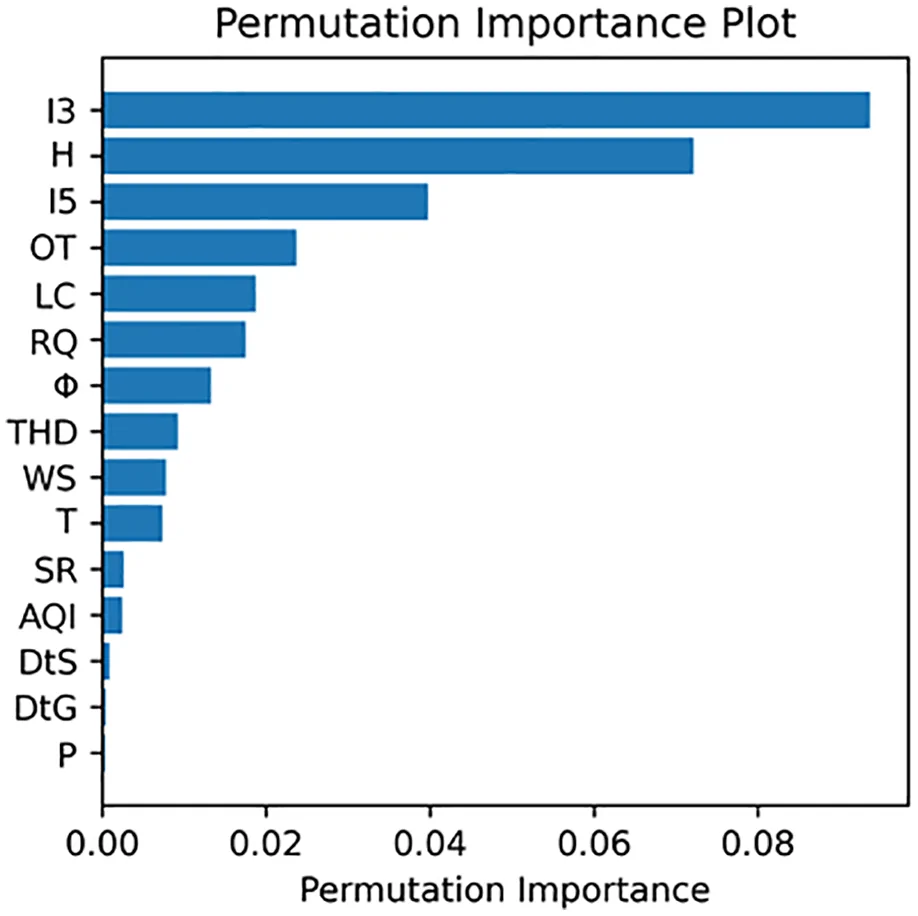
Features permutation importance.

To further assess the robustness of the permutation feature importance analysis was for all 15 features conducted, as shown in Fig 14 and Table 13. The results indicate that features such as ‘I3’, ‘H’, ‘I5’, and ‘OT’ consistently rank among the most important predictors, which is broadly in agreement with the SHAP-based findings across different models. Additional features, including ‘LC’, ‘RQ’, ‘THD’, and ‘WS’, also appear in both analyses, although their relative rankings vary. These differences are expected given the presence of strong multicollinearity among predictors, where correlated features may share or redistribute importance depending on the method used. While SHAP evaluates feature contributions within the model structure, permutation importance reflects the impact of feature perturbation on predictive performance. The general consistency between these approaches supports the relevance of key variables, while the observed discrepancies reinforce the need for cautious interpretation of feature importance in highly correlated settings.

**Table 13:** Top 10 features based on permutation importance.

Rank	Feature	Importance
1	I3	0.093627
2	H	0.072163
3	I5	0.039743
4	OT	0.02367
5	LC	0.018757
6	RQ	0.017507
7	Φ	0.01329
8	THD	0.009201
9	WS	0.007774
10	T	0.007336

### Model sensitivity to feature variations

**Fig 15:**
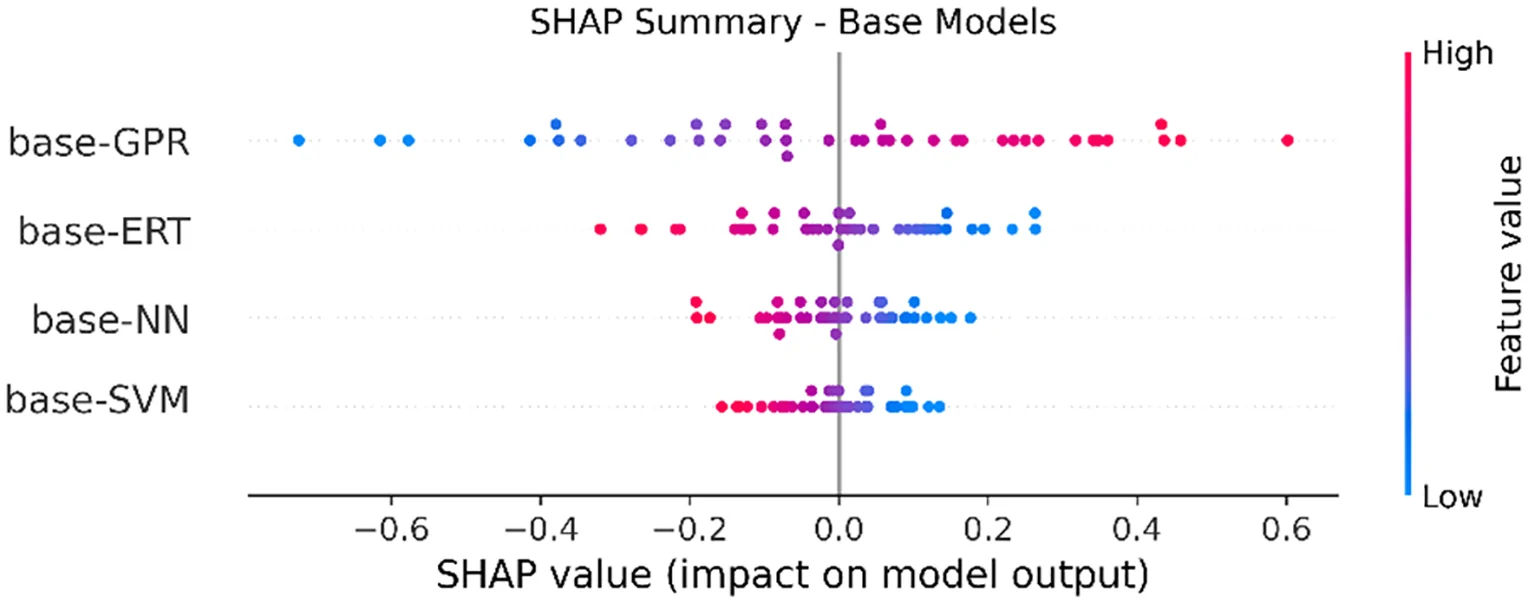
SHAP results-based methods impact.

Fig 15 presents SHAP summary plots illustrating model sensitivity to feature variations. The GPR model exhibits a wider range of SHAP values, suggesting greater sensitivity to input changes, while ETR and NN show more balanced responses. The SVM model displays relatively lower-magnitude SHAP values, indicating comparatively lower sensitivity. These observations reflect differences in how each model utilizes the input feature space rather than definitive differences in physical influence. Overall, SHAP provides valuable insight into model behavior and feature interactions; however, the results should be interpreted as complementary to other analyses rather than as conclusive evidence of physical importance.

### Effect of Feature Selection on the Performance of the Proposed Model

**Fig 16:**
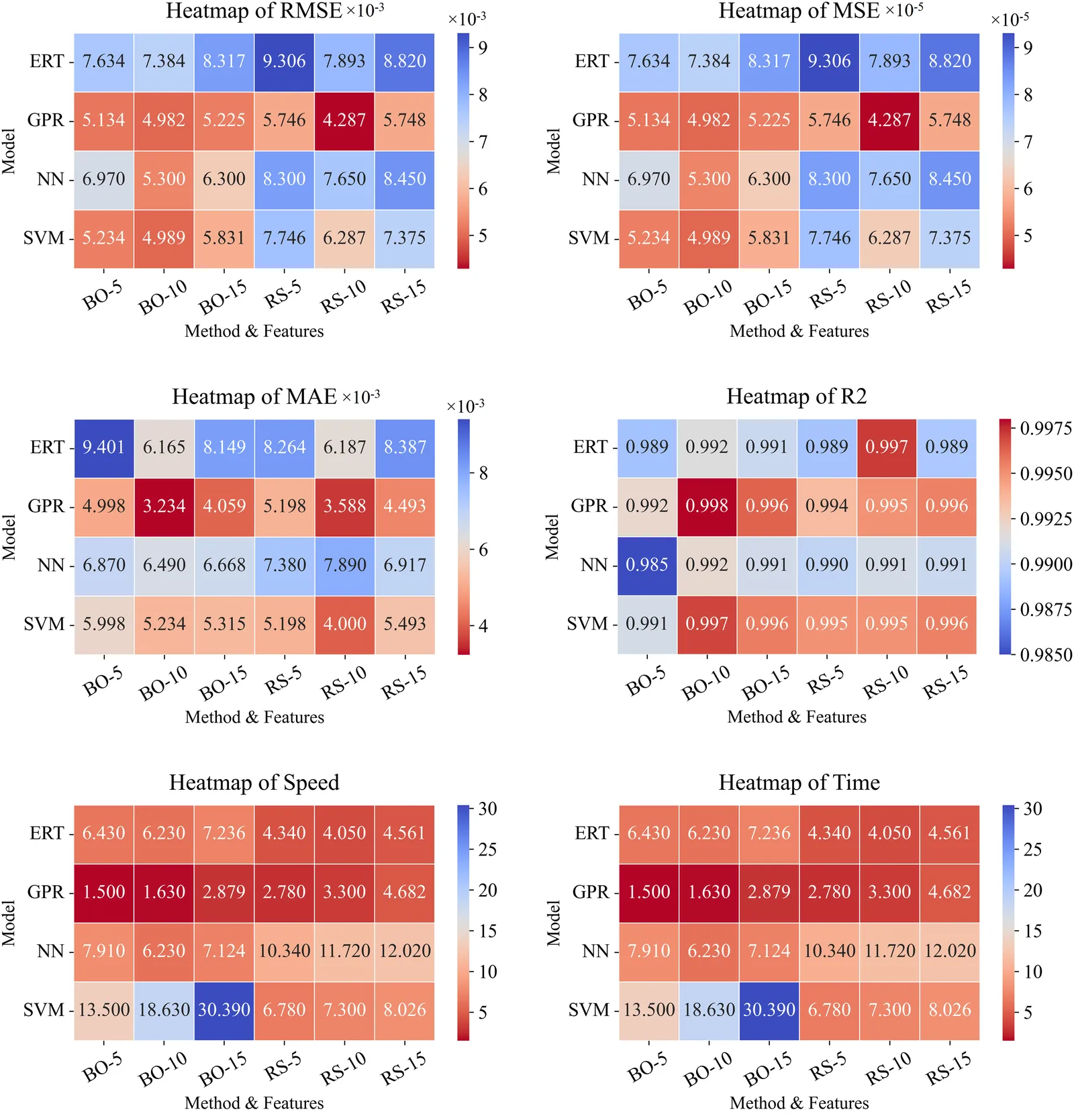
Metrics evaluation results for proposed models with 15, 10, and 5 top features based on SHAP results.

In predictive modelling, the performance and efficiency of machine learning (ML) algorithms are highly dependent on the selection of relevant input features. Feature selection not only improves model generalization but also reduces overfitting, computational complexity, and training time. In this study, we investigate the impact of feature selection on predictive performance by evaluating proposed machine learning models. The models are tested using three different feature configurations: (1) the full set of 15 features, (2) the top 10 features, and (3) the top 5 most influential features based on permutation importance value rankings. The evaluation is conducted using standard regression metrics, including RMSE, MSE, MAE, R^2^, prediction speed (P. speed), and training time. As shown in Fig 16, the results show that the 10-feature set offers the best trade-off between performance and simplicity. GPR benefits most, demonstrating improved accuracy and lower errors by effectively capturing nonlinear patterns when low-impact features are removed. ERT also performs better with 10 features, confirming its robustness with reduced yet informative inputs. Interestingly, the SVM model slightly improves with just 5 features, showing tolerance to dimensionality reduction, while NN maintains stable results across all configurations, reflecting its sensitivity to hyperparameters rather than feature count. Overall, the findings highlight the effectiveness of SHAP-based feature selection in enhancing model efficiency and interpretability, particularly for GPR and ERT, without compromising predictive power.

### Performance Comparison of Proposed and Baseline Models

**Fig 17:**
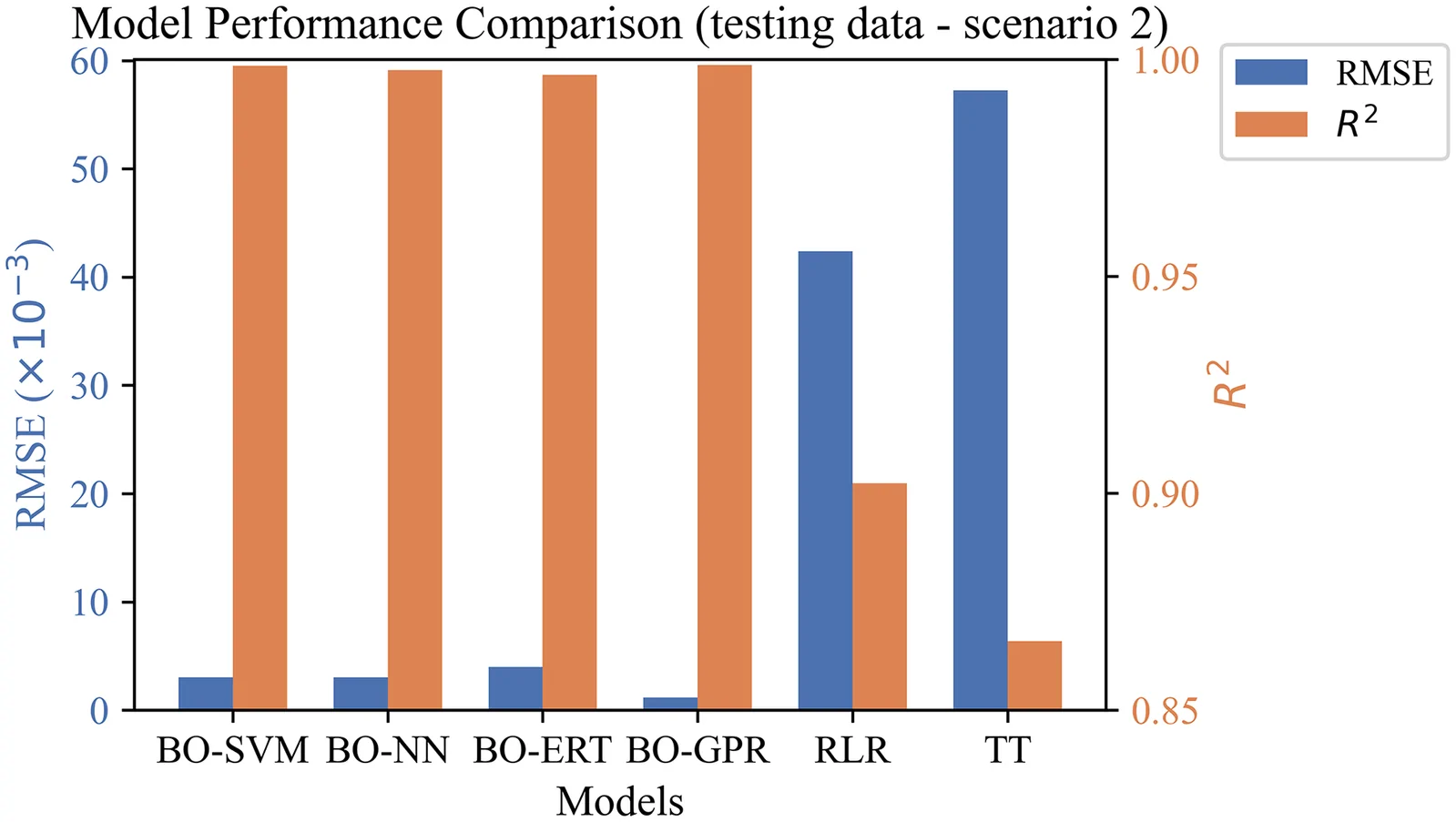
Comparison of the proposed model with baseline models (RLR, TT) for the testing dataset and 10 features.

The results presented in Fig 17 provide a comparative evaluation of the predictive performance of proposed models and sample baseline models such as regularized linear regression (RLR) and a transparent tree (TT) for testing datasets using RMSE and R^2^ as metrics. Among the models, the BO-optimized Gaussian Process Regression (GPR) with **scenario 2** splitting when using 10 features demonstrates the best overall performance, achieving the lowest RMSE (1.028 × 10 ⁻ ³), and highest 𝑅^2^ (0.9991), indicating superior accuracy and strong generalization capability. Support Vector Machine (SVM) and Neural Network (NN) models also show competitive performance, with relatively low RMSE values and high R^2^ scores. In contrast, simpler baseline models such as Regularized Linear Regression (RLR) and Decision Tree (DT) exhibit significantly higher RMSE and significantly lower R^2^, highlighting their limited ability to capture the underlying nonlinear relationships in the dataset.

## Conclusions

In this study, four machine learning algorithms, including Support Vector Machine (SVM), Neural Networks (NN), Extremely Randomized Trees (ERT), and Gaussian Process Regression (GPR), were employed to accurately predict the Equivalent Salt Deposit Density (ESDD) on high-voltage insulators, using real-world data collected from a project in Saudi Arabia. To enhance the performance of these models, hyperparameter tuning was performed using Bayesian Optimization (BO) and Random Search (RS). The models were evaluated based on several criteria, including prediction error metrics (RMSE, MSE, MAE, and MAPE), training duration, inference speed, and model size. The analysis was conducted using data collected over six months, with annual evaluations highlighting improvements in model accuracy, attributed to a better grasp of pollution behaviour across different seasons. The main findings are summarized as follows:

I. The application of the optimization techniques, such as BO and RS, clearly enhanced the performance of the proposed machine learning model.
II. The model that performs the best overall is the BO-optimized Gaussian Process Regression (GPR) with **scenario 2** splitting when utilizing 10 features. It has the lowest RMSE (1.028 × 10 ⁻ ³) and the greatest 𝑅^2^ (0.9991), suggesting higher accuracy and good generalization potential.
III. When comparing the two optimization techniques, BO generally yielded better model performance for most of the models due to its probabilistic strategy that efficiently navigates the hyperparameter space. However, for testing NN, RS performed better, suggesting that for certain models, random sampling can still identify strong configurations.
IV. SHAP improves transparency by explaining how different features impact ESDD predictions across machine learning models. The distinct patterns observed in SVM, NN, ERT, and GPR reflect each model’s unique approach to learning from data, showcasing differences in how they handle feature interactions, nonlinearity, and data structure to achieve accurate, interpretable outcomes.
V. SHAP-based feature selection greatly enhances model performance, with the 10-feature set providing the best balance between accuracy and simplicity. GPR and ERT show the greatest improvement, achieving higher accuracy and lower error with fewer, more impactful input features.

This research contributes to developing reliable pollution level prediction frameworks by integrating advanced ML models with optimization methods, aiding KSA’s transition toward sustainability in the electrical grid. Precise predictive models support improved forecasting, planning, and integration of high-voltage insulator safety into the national grid.

### Future work

Future work will focus on the practical deployment of the proposed predictive framework within real-world power system environments. In particular, the developed models can be integrated into SCADA and condition monitoring systems to enable real-time assessment of insulator contamination severity. This integration would allow utilities to perform predictive maintenance by identifying high-risk insulators before failure occurs. Furthermore, the scalability and generalizability of the model will be evaluated using larger datasets collected from different geographical regions and operating conditions. Additional efforts will also investigate the implementation of the framework in online monitoring platforms, enabling continuous data acquisition and automated decision support for high-voltage network maintenance.

## Supporting information

S1 File Proposed Machine learning description in detail. (PDF)

S1 Table Performance metrics results of the proposed models before optimization for all scenarios (mean±SD). (PDF)

S2 Table Optimized SVM Performance Metrics (Mean ± SD) Across All Scenarios. (PDF)

S3 Table Optimized NN Performance Metrics (Mean ± SD) Across All Scenarios. (PDF)

S4 Table Optimized ERT Performance Metrics (Mean ± SD) Across All Scenarios. (PDF)

S5 Table Optimized GPR Performance Metrics (Mean ± SD) Across All Scenarios. (PDF)
