# Supplementary material for: Machine learning framework for predicting ESDD on high-voltage glass insulators with SHAP-based feature optimization
Source: PLoS One. 2026 Aug 28;21(8):e0354892. doi: 10.1371/journal.pone.0354892 (PMC13524342; doi:10.1371/journal.pone.0354892)
Supplement: Supplementary file 1 [file pone.0354892.s001.pdf]

Proposed Machine learning description in detail

Neural Network (NN)

NN consists of an input layer, hidden layers, and an output layer that predicts ESDD. These layers are connected by artificial neurons with weights and biases controlling activation functions. Neurons process input signals using specific functions before transmitting the output to the next layer. This process allows the network to learn complex relationships within the data. The ANN input layer consists of N neurons, denoted as $X_1, X_2, \ldots\ldots, X_N$, where there are 15 input features. For the hidden layers l=1, 2, …, L−1, the output of each neuron is computed according to

$$Z_{lj} = W_{lj} \times A_{l-1} + b_{lj}, \quad A_{lj} = ReLU(Z_{lj}) \qquad \text{(s1)}$$

Continuous ESDD forecasts are made using Relu because of its computationally inexpensive and non-linear characteristics. The *ReLUa* function in neural networks is defined as *ReLU(a)=max(0, a)*. This means that when the input value a is positive, the function returns the input itself (*ReLU(a)* = *a*), while for non-positive values or *0*, it outputs zero, as shown in Fig s1. In the context of a neural network, the input to the jth neuron in the lth hidden layer is denoted as Z_{lj}. The weight matrix W_{lj} connects the neurons of the $(l-1)^{th}$ layer to the lth hidden layer, and A_{l-1} represents the output from the (l−1)th layer, which serves as the input to the lth layer. Additionally, b_{lj} refers to the bias term associated with the jth neuron in the lth hidden layer. The prediction of ESDD occurs at the output layer, labeled as the Lth layer. Here, the computation of the input to the kth neuron in the output layer is given by

$$Z_{Lk} = W_{Lk} \times A_{L-1} + b_{Lk} \qquad \text{(s2)}$$

The predicted ESDD is directly represented by $A_{Lk} = Z_{Lk}$. During the training phase, optimization techniques such as gradient descent or backpropagation are employed to fine-tune the weights (W_{lj}, W_{Lk}) and biases (b_{lj}, b_{Lk}), aiming to minimize the discrepancy between the predicted and actual ESDD values.

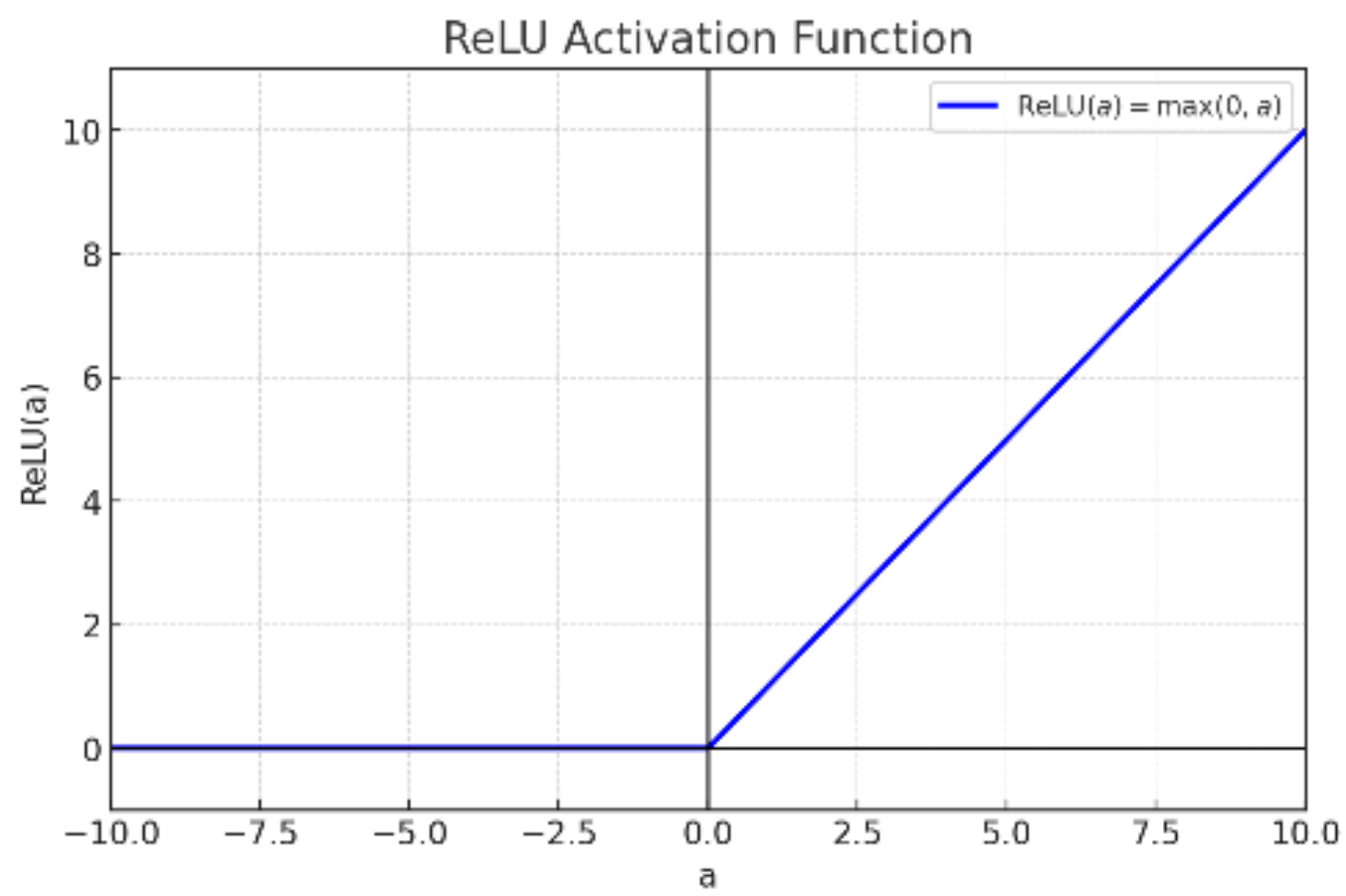

Fig s1. ReLU graphical depiction.

Extremely Randomized Trees (ERT)

The study investigates ensemble learning techniques to enhance prediction accuracy and model resilience within ensemble learning. It emphasizes two primary ensemble strategies: bagging and boosting. Bagging entails training decision trees independently on bootstrapped data, which reduces variance and boosts generalization.

Conversely, boosting employs a sequential method to correct errors made by prior trees, concentrating on difficult cases. Both techniques significantly enhance ESDD forecast accuracy. In an ensemble model using ERT, the algorithm builds M decision trees $[Tr_1, Tr_2, \ldots\ldots, Tr_M]$, each trained on a random subset of the training data. For a dataset with N samples, input features are represented as $U = [u_1, u_2, \ldots\ldots, u_N]$, and target values as $V = [v_1, v_2, \ldots\ldots, v_N]$. The prediction for an input vector *x* is computed as,

$$F(x) = \sum f_i(u) \tag{s3}$$

where $f_i(u)$ is the prediction from the ith tree. Training each tree involves three steps: Random Sampling “A bootstrap sample is drawn, allowing some samples to appear multiple times or not at all”; Tree Construction “Trees are built recursively by selecting the best feature and split point using criteria like Gini index or information gain until a stopping condition is met”; Aggregation “Predictions from all trees are combined—averaged for regression (bagging) or weighted voting for

classification (boosting)". Mathematically, the prediction of the ith tree is $f_i(u) = Tr_i(u)$, and the final ERT prediction combines outputs from all trees in the ensemble.

Gaussian Process Regression (GPR)

GPR is a probabilistic machine learning technique utilized for regression tasks, such as predicting ESDD. It relies on Gaussian processes (GP), which estimate unknown functions using observed data, defined by a mean function and a covariance function (kernel). Common kernels include squared exponential, Matérn, and exponential [1]. In this work, GPR predicts ESDD by defining priority, collecting meteorological and electrical data, computing covariance, adding noise, calculating posterior distributions, making predictions, and optimizing hyperparameters using Bayesian Optimization and Random Search. Given a training dataset $D = \{(c_i, d_i)|i = 1, \dots, n\}$, where each pair consists of an input $c_i$and its corresponding output d_i, GPR can be used to predict the output $d_{new}$for a new input c_{new}. Specifically, a linear GPR model employs a linear kernel to capture relationships between variables, enabling precise predictions [2] as,

$$d = c^T\beta + \mu \tag{s4}$$

where $\mu \sim N(0, \sigma_n^2)$ represents a noise in the observations. This noise is characterized by a Gaussian distribution with a mean of 0 and variance σ_n^2. The regression process involves estimating the coefficients $\beta$and the noise varianceσ_n^2using the provided data. In GPR, the solution incorporates latent variables $\{f(c_i)|i = 1, \dots, n\}$and basic functions h, transforming the model into (s5),

$$d = h(c^T)\beta + f(c) \tag{s5}$$

here $f(c)$ is modeled as a Gaussian Process with zero mean and a covariance function $k(x, x')$The probabilistic GPR framework yields solutions where the likelihood of the observed outputs $d_i$given$f(c_i)$ and c_i follows a normal distribution as,

$$P(d_{i|} \mid f(c_i), c_i) \sim N(d_{i|} \mid h(c_i)^T\beta + f(c_i), \sigma^2) \tag{s6}$$

Consequently, the joint distribution of the latent variables in the GPR model can be expressed as,

$$P(f|C) \sim N(f|0, K(C, C')) \tag{s7}$$

$$C = [c_1^T, c_2^T, \dots, c_n^T] \tag{s8}$$

$$K(C,C') = \begin{bmatrix} k(c_1,c_1) & k(c_1,c_2) & \cdots & k(c_1,c_n) \\ k(c_1,c_2) & k(c_2,c_2) & \cdots & k(c_2,c_n) \\ \vdots & \vdots & \ddots & \vdots \\ k(c_n,c_1) & k(c_n,c_2) & \cdots & k(c_n,c_n) \end{bmatrix} \tag{s9}$$

C denotes the input data matrix. The hyperparameters to optimize for the mentioned machine learning (ML) methods, including their range values are shown *i*.

Optimizers Algorithms

Random Search Optimizations

Random Search (RS) and Grid Search (GS) are two widely used techniques for hyperparameter optimization. In RS, hyperparameters are randomly sampled from a predefined distribution to explore the search space, whereas GS systematically evaluates all possible hyperparameter combinations. Fig s2 illustrates the working principles of GS and RS using two hyperparameters of Extremely Randomized Trees (ERT): the number of predictors and the minimum leaf size. RS selects random values for both hyperparameters, ensuring efficient coverage of the search space. Conversely, GS evaluates all possible configurations exhaustively. For instance, GS would analyse all combinations of 10 predictors with different leaf sizes, making it computationally impractical for large datasets with numerous hyperparameters. RS offers a more efficient exploration strategy, represented mathematically as

$$q^* = argmin_{x\in\Omega} f(q) \tag{s10}$$

where Ω represents the total set of possible hyperparameter values, which given by,

$$\Omega = \{(a_1,b_1,...,z_1),(a_2,b_2,...,z_2),...,(a_n,b_n,...,z_n)\} \tag{s11}$$

Each configuration is evaluated on a validation dataset using

$$q^* = argmin_{x\in\Omega} f(q,\psi^*;S_{validation}) \tag{s12}$$

For RS, the optimization is expressed as,

$$\delta^* = argmin_{\delta\in C} f(q,\psi;S_{train}) \tag{s13}$$

where ψ represents the learned hyperparameters. RS is particularly effective when dealing with large search spaces or when there is limited prior knowledge about promising hyperparameter

values. It often outperforms GS in terms of efficiency, as its randomized approach can uncover optimal configurations that GS might miss within a limited number of trials.

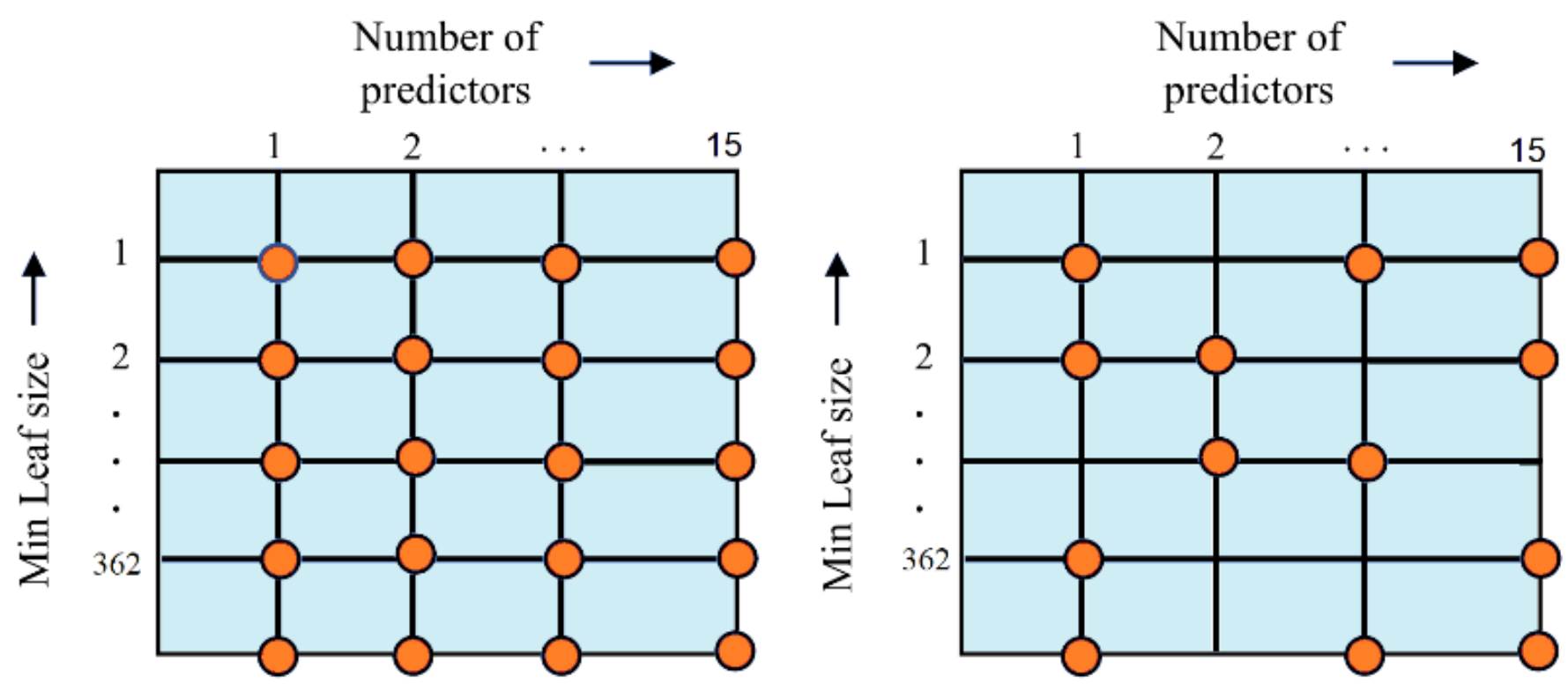

(a) (b)

Fig s2. Tree algorithm hyperparameter optimization (a) Grid search, (b) Random search**.**

Bayesian Optimization

RS and GS optimizers often explore the entire hyperparameter search space without utilizing past evaluations, leading to inefficiency. Bayesian Optimization (BO) uses an iterative approach, using past results to model the objective function. As observations accumulate, BO becomes more adept at identifying promising regions, resulting in better model settings in fewer iterations. BO selects the next sampling point by maximizing a reward function, aiming to locate the global optimum of complex, nonconvex, and black-box functions. Mathematically, BO seeks to minimize the objective function $f(g)$ over a bounded sample space GD with D dimensions as,

$$g^* = \arg\min_{g \in GD} f(g) \tag{s14}$$

where g is the input vector representing control parameters for optimization, and $\tilde{g}$ denotes the global optimum. This methodology is grounded in Bayes' Theorem as,

$$Q(s \mid E_{1:t}, \theta) = \left(Q(E_{1:t} \mid h, \theta) Q(h \mid, \theta)\right) / \left(Q(E_{1:t} \mid \theta)\right) \tag{s15}$$

where prior, likelihood, and posterior distributions guide the search process. Specifically, noisy observations $h_i = f(g_i) + \epsilon_i$ of the objective function are used to update the posterior distribution after each iteration t. The dataset $E_{1:t} = \{(g_1, h_1), (g_2, h_2), \ldots, (g_t, h_t)\}$ accumulates observations, and the posterior $Q(h \mid E_{1:t}, \theta)$ refines the model's understanding of promising regions. By focusing on the

most promising areas, BO efficiently narrows down the search space, making it a powerful tool for hyperparameter optimization. BO leverages Gaussian Processes (GPs) as a performance model to represent a distribution over possible functions. The GP is defined by a mean function $m(g)$, which estimates the expected value of the target function at each point in the parameter space, and a covariance function $e(g,g')$, which captures the uncertainty or variability between function values at different points. Mathematically, this is expressed as,

$$f(g) = GP(m(g), e(g, g')) \tag{s16}$$

where $f(g)$ represents the function value at point g. As shown in Fig 5, the optimization process is guided by an acquisition function, which balances exploration (searching unexplored regions) and exploitation (focusing on high-performing regions). The acquisition function evaluates the potential utility of each point in the parameter space and determines the next sampling point. It is mathematically represented as,

$$AQ(x) = acq(m(g), sd(g)) \tag{s17}$$

where $AQ(x)$ depends on the mean $m(g)$ and standard deviation $sd(g)$, which quantify the expected performance and uncertainty, respectively. The Probability of Improvement (PI), Expected Improvement (EI), and Upper Confidence Bound (UCB) are common acquisition functions that offer a trade-off between exploration and exploitation. The iterative BO process evaluates performance models, updates the GP with observed data, and selects the next sampling point, efficiently narrowing the search space to locate the global optimum, making it effective for complex optimization problems.

[1] Y. Deng, Y. Zheng, Z. Meng, H. Gao, Y. Hua, Q. Jin, Gaussian process regression for evolutionary dynamic multiobjective optimization in complex environments, Swarm Evol. Comput. 94 (2025) 101883. https://doi.org/10.1016/j.swevo.2025.101883.

[2] E. Schulz, M. Speekenbrink, A. Krause, A tutorial on Gaussian process regression: Modelling, exploring, and exploiting functions, J. Math. Psychol. 85 (2018) 1–16. https://doi.org/10.1016/j.jmp.2018.03.001.
