# Supplementary material for: Machine learning framework for predicting ESDD on high-voltage glass insulators with SHAP-based feature optimization
Source: PLoS One. 2026 Aug 28;21(8):e0354892. doi: 10.1371/journal.pone.0354892 (PMC13524342; doi:10.1371/journal.pone.0354892)
Supplement: Supplementary file 2 [file pone.0354892.s002.pdf]

S1 Table. Performance metrics results of the proposed models before optimization for all scenarios (mean ± SD).

Model	RMSE $\times 10^{-3}$	MSE $\times 10^{-6}$	MAE $\times 10^{-3}$	R^2	MAPE%
Scenario 1 - Training					
SVM	13.43 ± 0.82	180.48 ± 6.75	11.27 ± 0.64	0.9756 ± 0.021	4.7 ± 0.33
NN	15.29 ± 0.91	233.89 ± 7.12	13.51 ± 0.72	0.9684 ± 0.025	5.1 ± 0.42
ERT	25.66 ± 1.34	658.48 ± 12.8	21.01 ± 1.05	0.9111 ± 0.038	8.8 ± 0.61
GPR	12.52 ± 0.76	165.91 ± 5.98	9.14 ± 0.52	0.9788 ± 0.019	3.7 ± 0.25
Scenario 1 - Testing					
SVM	11.87 ± 0.69	140.97 ± 5.43	10.47 ± 0.58	0.9707 ± 0.024	5.2 ± 0.41
NN	14.55 ± 0.88	211.82 ± 6.87	12.91 ± 0.67	0.9610 ± 0.028	5.6 ± 0.47
ERT	27.97 ± 1.42	782.52 ± 14.3	23.56 ± 1.12	0.8930 ± 0.041	11.1 ± 0.72
GPR	6.12 ± 0.48	137.50 ± 4.21	5.28 ± 0.37	0.9718 ± 0.023	5.2 ± 0.49
Scenario 2 - Training					
SVM	10.634 ± 0.42	114.77 ± 6.89	5.762 ± 0.29	0.9825 ± 0.018	5.23 ± 0.31
NN	12.557 ± 0.50	206.14 ± 10.31	11.471 ± 0.57	0.9775 ± 0.021	5.61 ± 0.34
ERT	15.644 ± 0.78	587.95 ± 23.52	12.873 ± 0.64	0.9371 ± 0.030	7.93 ± 0.48
GPR	10.375 ± 0.41	107.43 ± 5.91	5.198 ± 0.26	0.9842 ± 0.017	4.13 ± 0.25
Scenario 2 - Testing					
SVM	11.88 ± 0.71	109.51 ± 5.48	8.66 ± 0.52	0.9784 ± 0.021	4.02 ± 0.24
NN	12.07 ± 0.72	201.54 ± 10.08	10.71 ± 0.64	0.9638 ± 0.027	3.68 ± 0.22
ERT	13.45 ± 0.81	589.46 ± 29.47	12.22 ± 0.73	0.9364 ± 0.033	6.92 ± 0.42
GPR	10.88 ± 0.65	99.72 ± 4.99	5.63 ± 0.34	0.9802 ± 0.020	3.69 ± 0.22
Scenario 3 - Training					
SVM	11.23 ± 0.45	146.50 ± 7.33	9.14 ± 0.46	0.9779 ± 0.020	6.10 ± 0.37
NN	13.67 ± 0.55	234.91 ± 11.75	13.35 ± 0.67	0.9632 ± 0.025	6.40 ± 0.38
ERT	22.38 ± 1.12	699.12 ± 27.96	14.76 ± 0.74	0.9260 ± 0.033	9.20 ± 0.55
GPR	12.34 ± 0.49	146.23 ± 7.31	7.75 ± 0.39	0.9793 ± 0.019	4.50 ± 0.27
Scenario 3 - Testing					
SVM	12.37 ± 0.74	136.13 ± 6.81	12.23 ± 0.73	0.9747 ± 0.022	4.13 ± 0.25
NN	14.94 ± 0.90	248.46 ± 12.42	15.13 ± 0.91	0.9621 ± 0.028	4.45 ± 0.27
ERT	25.63 ± 1.54	686.65 ± 34.33	19.28 ± 1.16	0.9099 ± 0.039	7.21 ± 0.43
GPR	14.03 ± 0.84	181.76 ± 9.09	7.71 ± 0.46	0.9760 ± 0.021	3.87 ± 0.23
Scenario 4 - Training					
SVM	11.56 ± 0.7	178.78 ± 6.1	10.42 ± 0.6	0.9761 ± 0.022	6.2 ± 0.42
NN	13.12 ± 0.8	247.57 ± 7.4	13.68 ± 0.7	0.9662 ± 0.025	6.4 ± 0.45
ERT	27.56 ± 1.4	714.34 ± 15.3	18.89 ± 1.0	0.9136 ± 0.039	10.2 ± 0.67
GPR	10.41 ± 0.6	160.13 ± 5.7	7.83 ± 0.5	0.9791 ± 0.018	4.5 ± 0.39
Scenario 4 - Testing					
SVM	13.37 ± 0.8	184.87 ± 6.1	12.44 ± 0.7	0.9736 ± 0.022	4.4 ± 0.38
NN	15.26 ± 0.9	255.11 ± 7.8	15.19 ± 0.8	0.9601 ± 0.026	4.5 ± 0.36
ERT	29.44 ± 1.5	726.78 ± 14.9	19.32 ± 1.1	0.9086 ± 0.037	7.6 ± 0.52
GPR	14.54 ± 0.9	189.31 ± 6.3	7.96 ± 0.5	0.9749 ± 0.021	4.9 ± 0.34
