# Supplementary material for: Machine learning framework for predicting ESDD on high-voltage glass insulators with SHAP-based feature optimization
Source: PLoS One. 2026 Aug 28;21(8):e0354892. doi: 10.1371/journal.pone.0354892 (PMC13524342; doi:10.1371/journal.pone.0354892)
Supplement: Supplementary file 3 [file pone.0354892.s003.pdf]

S2 Table. **SVM Performance Metrics (Mean ± SD) Across All Scenarios**

Phase	Opt	RMSE $\times10^{-3}$	MSE $\times10^{-6}$	MAE $\times10^{-3}$	R^2	MAPE%
**Scenario 1**						
SVM Train	RS	7.37 ± 0.42	5.439 ± 0.21	4.493 ± 0.25	0.9955 ± 0.012	2.3 ± 0.28
	BO	5.83 ± 0.35	3.435 ± 0.18	4.315 ± 0.23	0.9956 ± 0.011	2.0 ± 0.26
SVM Test	RS	8.78 ± 0.51	1.434 ± 0.09	5.568 ± 0.31	0.9980 ± 0.008	1.3 ± 0.19
	BO	4.21 ± 0.33	1.173 ± 0.08	3.204 ± 0.22	0.99857 ± 0.007	1.1 ± 0.17
**Scenario 2**						
SVM Train	RS	5.57 ± 0.33	2.899 ± 0.14	3.312 ± 0.18	0.9973 ± 0.009	1.80 ± 0.18
	BO	2.47 ± 0.19	2.938 ± 0.12	3.198 ± 0.17	0.9881 ± 0.012	1.60 ± 0.15
SVM Test	RS	2.67 ± 0.21	0.877 ± 0.05	3.565 ± 0.20	0.9984 ± 0.006	0.59 ± 0.07
	BO	2.86 ± 0.22	0.794 ± 0.04	2.086 ± 0.14	0.9990 ± 0.005	0.487 ± 0.05
**Scenario 3**						
SVM Train	RS	6.23 ± 0.37	3.963 ± 0.18	4.256 ± 0.22	0.9960 ± 0.010	1.80 ± 0.18
	BO	3.65 ± 0.25	3.224 ± 0.16	3.982 ± 0.21	0.9966 ± 0.009	1.70 ± 0.17
SVM Test	RS	3.12 ± 0.24	1.356 ± 0.07	4.872 ± 0.26	0.9983 ± 0.007	0.68 ± 0.08
	BO	3.04 ± 0.23	1.029 ± 0.06	2.146 ± 0.15	0.9986 ± 0.006	0.603 ± 0.06
**Scenario 4**						
SVM Train	RS	6.37 ± 0.38	4.97 ± 0.21	4.311 ± 0.24	0.9958 ± 0.010	2.1 ± 0.22
	BO	3.83 ± 0.29	3.36 ± 0.18	4.146 ± 0.23	0.9963 ± 0.009	1.9 ± 0.13
SVM Test	RS	3.32 ± 0.25	1.39 ± 0.07	5.268 ± 0.28	0.9982 ± 0.006	0.76 ± 0.08
	BO	3.08 ± 0.24	1.038 ± 0.06	2.902 ± 0.18	0.9987 ± 0.005	0.623 ± 0.06
