# Supplementary material for: Machine learning framework for predicting ESDD on high-voltage glass insulators with SHAP-based feature optimization
Source: PLoS One. 2026 Aug 28;21(8):e0354892. doi: 10.1371/journal.pone.0354892 (PMC13524342; doi:10.1371/journal.pone.0354892)
Supplement: Supplementary file 4 [file pone.0354892.s004.pdf]

S3 Table. Optimized NN Performance Metrics (Mean ± SD) Across All Scenarios.

Phase	Opt	RMSE $\times10^{-3}$	MSE $\times10^{-6}$	MAE $\times10^{-3}$	R^2	MAPE%
**Scenario 1**						
NN Train	RS	8.317 ± 0.50	6.917 ± 0.28	6.149 ± 0.34	0.9906 ± 0.015	2.7 ± 0.2
	BO	8.166 ± 0.48	6.668 ± 0.26	6.127 ± 0.33	0.9909 ± 0.014	2.6 ± 0.2
NN Test	RS	4.656 ± 0.36	2.168 ± 0.11	3.495 ± 0.21	0.99703 ± 0.009	1.6 ± 0.18
	BO	5.362 ± 0.40	2.876 ± 0.13	4.153 ± 0.24	0.99606 ± 0.010	1.8 ± 0.14
**Scenario 2**						
NN Train	RS	5.725 ± 0.34	4.828 ± 0.19	3.648 ± 0.20	0.9924 ± 0.014	2.2 ± 0.21
	BO	3.864 ± 0.27	4.172 ± 0.17	3.053 ± 0.18	0.9937 ± 0.013	1.7 ± 0.17
NN Test	RS	2.331 ± 0.18	1.877 ± 0.08	3.264 ± 0.19	0.9986 ± 0.008	0.90 ± 0.09
	BO	2.965 ± 0.22	1.216 ± 0.06	3.518 ± 0.20	0.9978 ± 0.009	1.08 ± 0.10
**Scenario 3**						
NN Train	RS	6.557 ± 0.39	5.895 ± 0.24	4.252 ± 0.23	0.9912 ± 0.015	2.4 ± 0.23
	BO	4.987 ± 0.31	4.405 ± 0.19	4.032 ± 0.22	0.9928 ± 0.014	2.3 ± 0.21
NN Test	RS	2.366 ± 0.18	2.027 ± 0.09	3.325 ± 0.19	0.9984 ± 0.008	1.2 ± 0.11
	BO	3.019 ± 0.23	2.526 ± 0.11	4.100 ± 0.23	0.9976 ± 0.009	1.3 ± 0.12
**Scenario 4**						
NN Train	RS	6.687 ± 0.42	5.944 ± 0.24	4.491 ± 0.26	0.9909 ± 0.012	2.6 ± 0.27
	BO	5.773 ± 0.38	4.686 ± 0.21	4.172 ± 0.24	0.9912 ± 0.011	2.4 ± 0.24
NN Test	RS	2.566 ± 0.21	2.145 ± 0.09	3.459 ± 0.19	0.9980 ± 0.007	1.5 ± 0.11
	BO	3.623 ± 0.28	2.761 ± 0.11	4.103 ± 0.23	0.99701 ± 0.008	1.6 ± 0.10
