# Supplementary material for: Machine learning framework for predicting ESDD on high-voltage glass insulators with SHAP-based feature optimization
Source: PLoS One. 2026 Aug 28;21(8):e0354892. doi: 10.1371/journal.pone.0354892 (PMC13524342; doi:10.1371/journal.pone.0354892)
Supplement: Supplementary file 5 [file pone.0354892.s005.pdf]

S4 Table. Optimized ERT Performance Metrics (Mean ± SD) Across All Scenarios.

Phase	Opt	RMSE $\times 10^{-3}$	MSE $\times 10^{-6}$	MAE $\times 10^{-3}$	R^2	MAPE%
Scenario 1						
ERT Train	RS	8.820 ± 0.52	7.779 ± 0.30	6.387 ± 0.35	0.9894 ± 0.014	2.7 ± 0.22
	BO	8.317 ± 0.49	6.917 ± 0.28	6.149 ± 0.34	0.9906 ± 0.013	2.7 ± 0.21
ERT Test	RS	5.196 ± 0.38	2.700 ± 0.12	4.025 ± 0.23	0.9963 ± 0.009	1.8 ± 0.16
	BO	4.656 ± 0.34	2.168 ± 0.10	3.495 ± 0.21	0.9970 ± 0.008	1.7 ± 0.11
Scenario 2						
ERT Train	RS	4.843 ± 0.29	2.973 ± 0.12	4.238 ± 0.23	0.9912 ± 0.018	2.1 ± 0.17
	BO	4.396 ± 0.27	2.845 ± 0.11	4.319 ± 0.24	0.9918 ± 0.017	2.3 ± 0.19
ERT Test	RS	4.365 ± 0.30	2.034 ± 0.08	3.251 ± 0.18	0.9972 ± 0.012	1.5 ± 0.12
	BO	3.695 ± 0.26	0.972 ± 0.05	2.594 ± 0.15	0.9978 ± 0.013	1.4 ± 0.13
Scenario 3						
ERT Train	RS	7.116 ± 0.43	6.814 ± 0.27	4.591 ± 0.25	0.9898 ± 0.016	2.2 ± 0.22
	BO	7.573 ± 0.45	6.012 ± 0.25	5.882 ± 0.31	0.9915 ± 0.017	2.8 ± 0.24
ERT Test	RS	4.925 ± 0.34	2.281 ± 0.10	3.921 ± 0.22	0.9961 ± 0.011	1.6 ± 0.14
	BO	3.993 ± 0.28	1.695 ± 0.08	2.890 ± 0.17	0.9965 ± 0.012	1.4 ± 0.12
Scenario 4						
ERT Train	RS	7.280 ± 0.44	7.191 ± 0.29	5.873 ± 0.32	0.9897 ± 0.013	2.5 ± 0.24
	BO	7.713 ± 0.46	6.271 ± 0.27	5.941 ± 0.33	0.9913 ± 0.012	2.6 ± 0.23
ERT Test	RS	5.104 ± 0.36	2.404 ± 0.11	3.952 ± 0.22	0.9966 ± 0.009	1.9 ± 0.18
	BO	4.065 ± 0.30	1.868 ± 0.09	3.594 ± 0.20	0.9980 ± 0.007	1.6 ± 0.11
