# Supplementary material for: Machine learning framework for predicting ESDD on high-voltage glass insulators with SHAP-based feature optimization
Source: PLoS One. 2026 Aug 28;21(8):e0354892. doi: 10.1371/journal.pone.0354892 (PMC13524342; doi:10.1371/journal.pone.0354892)
Supplement: Supplementary file 6 [file pone.0354892.s006.pdf]

S5 Table. Optimized GPR Performance Metrics (Mean ± SD) Across All Scenarios

Model	Opt.	RMSE	MSE	MAE	R^2	MAPE%
Scenario 1						
GPR Training	RS	5.748 ± 0.76	3.304 ± 0.42	4.493 ± 0.55	0.9955 ± 0.012	1.9 ± 0.31
	BO	5.225 ± 0.61	2.729 ± 0.38	4.059 ± 0.49	0.9963 ± 0.010	1.7 ± 0.24
GPR Testing	RS	3.671 ± 0.44	1.347 ± 0.21	2.786 ± 0.36	0.9981 ± 0.008	1.2 ± 0.22
	BO	2.699 ± 0.39	0.728 ± 0.15	1.891 ± 0.28	0.9983 ± 0.007	0.8 ± 0.12
Scenario 2						
GPR Training	RS	7.35 ± 0.92	5.36 ± 0.64	6.24 ± 0.71	0.9911 ± 0.018	2.2 ± 0.30
	BO	7.24 ± 0.85	5.19 ± 0.60	5.962 ± 0.68	0.9899 ± 0.020	2.4 ± 0.42
GPR Testing	RS	6.75 ± 0.73	4.74 ± 0.55	4.815 ± 0.60	0.9977 ± 0.010	1.9 ± 0.34
	BO	4.82 ± 0.58	3.81 ± 0.49	4.023 ± 0.52	0.9980 ± 0.009	1.1 ± 0.27
Scenario 3						
GPR Training	RS	5.86 ± 0.70	4.71 ± 0.58	5.912 ± 0.66	0.9935 ± 0.015	2.1 ± 0.35
	BO	4.91 ± 0.63	4.28 ± 0.51	4.989 ± 0.59	0.9919 ± 0.017	1.8 ± 0.22
GPR Testing	RS	4.46 ± 0.52	2.59 ± 0.33	3.743 ± 0.48	0.9978 ± 0.009	1.3 ± 0.28
	BO	3.62 ± 0.47	2.23 ± 0.29	3.312 ± 0.41	0.9981 ± 0.008	1.0 ± 0.15
Scenario 4						
GPR Training	RS	4.847 ± 0.59	3.005 ± 0.40	4.251 ± 0.51	0.9962 ± 0.011	1.6 ± 0.21
	BO	4.252 ± 0.54	2.126 ± 0.31	3.795 ± 0.45	0.9978 ± 0.009	1.4 ± 0.26
GPR Testing	RS	2.762 ± 0.36	1.276 ± 0.19	2.088 ± 0.30	0.9983 ± 0.007	1.1 ± 0.25
	BO	1.239 ± 0.28	0.688 ± 0.12	1.494 ± 0.22	0.9986 ± 0.006	0.6 ± 0.19
